# Enhanced sucrose production by controlling carbon flux through CfrA expression in *Synechocystis* sp. PCC 6803

**DOI:** 10.1186/s12934-025-02894-8

**Published:** 2025-12-31

**Authors:** María Teresa Domínguez-Lobo, Pablo Ortega-Martínez, Francisco J. Florencio, M. Isabel Muro-Pastor

**Affiliations:** Instituto de Bioquímica Vegetal y Fotosíntesis, CSIC-Universidad de Sevilla, 41092 Seville, Spain

**Keywords:** Sucrose, *Synechocystis*, CfrA, Glycogen, Carbon metabolism

## Abstract

**Background:**

Cyanobacteria, as phototrophic organisms with low nutritional requirements and great metabolic versatility, are attractive for the sustainable production of value-added chemicals from CO_2_ and sunlight. One limitation of these strategies is that carbon is partitioned towards biomass synthesis rather than product synthesis. An alternative to conventional metabolic engineering approaches involves controlling regulatory circuits to enhance the flow of carbon towards the synthesis of desired compounds. The carbon-flow-regulator A (CfrA) is pivotal in redirecting carbon flux during nitrogen deficiency in cyanobacteria, promoting glycogen accumulation by inhibiting 2,3-phosphoglycerate mutase enzyme. The moderately halotolerant cyanobacterium *Synechocystis* sp. PCC 6803 accumulates sucrose and glucosylglycerol (GG) as compatible solutes under salt stress. Sucrose is a valuable carbon source for heterotrophic organisms, whether they are cultivated independently or in co-cultures. In this context, we explored the potential biotechnological relevance of CfrA in redirecting carbon flow towards sucrose production.

**Results:**

A strain that overexpresses *cfrA*, independently of nitrogen growth conditions, and carries a plasmid that expresses sucrose-phosphate synthase (SPS) from *Synechocystis* sp. PCC 6803 and the heterologous sucrose permease CscB inducibly (P_*ars*_*-cfrA*/suc strain) was constructed and analysed. In this strain, *cfrA* expression increased sucrose production by 40% compared to non-induced levels. The fixed carbon was partially redirected towards sucrose production at the expense of glycogen accumulation and biomass generation. Furthermore, an improvement in the photosynthetic activity of this strain was observed due to the presence of this carbon sink. The effect of eliminating GG synthesis (Δ*ggpS*/P_*ars*_*-cfrA*/suc strain) on sucrose production was also analyzed. Under high salinity conditions (400 mM NaCl), this strain exhibited a maximum sucrose accumulation of 2.72 g/L. Encapsulation of the P_*ars*_*-cfrA*/suc strain has also been studied.

**Conclusions:**

Our results indicate that modulating carbon flow through CfrA overexpression can substantially boost sucrose production. Glycogen accumulation, mediated by CfrA, enhances sucrose production, which is partly derived from the use of stored glycogen. Furthermore, immobilising *Synechocystis* cells in alginate improves sucrose production and facilitates its utilisation. Given the widespread occurrence of the *cfrA* gene in cyanobacteria, its potential as a target in various biotechnological strategies that require the redirection of carbon flow should be considered.

**Supplementary Information:**

The online version contains supplementary material available at 10.1186/s12934-025-02894-8.

## Background

In the context of the demand for a sustainable economy that limits CO_2_ emissions and does not depend on fossil fuels, cyanobacteria have aroused great interest in recent years. As oxygenic photosynthetic organisms they are able to efficiently transform CO_2_ into organic carbon using solar energy and water [1]. Their high metabolic plasticity allows them to utilize inorganic carbon for producing a variety of valuable compounds. Although many cyanobacteria-based (photo)biotechnology strategies have been addressed, different challenges remain to achieve industrial-scale efficiency. In general, detailed knowledge of metabolism and its regulatory circuits is less developed than in other microorganisms of biotechnological interest [2]. However, recent advances include the development of new tools and specific technologies (expression vectors, omics approaches, metabolic modelling, etc.) as well as the characterization of highly productive, fast-growing strains. Likewise, emerging research into co-cultivation strategies involving cyanobacteria and other organisms of interest is significantly enhancing the robustness of many production processes [3].

The model cyanobacterium *Synechocystis* sp. PCC 6803 (hereinafter *Synechocystis*) exhibits a flexible carbon metabolism, is genetically tractable and moderately salt tolerant [1, 4, 5].

In addition to conventional approaches, based on the introduction of genes necessary to implement metabolic pathways, cyanobacterial engineering currently includes the development of customized tools for certain products and strains. These strategies include the modulation of intrinsic regulatory mechanisms, allowing action on metabolic fluxes and re-directing them towards specific routes and products. In this sense, recent studies highlight the role of several regulatory proteins as possible biotechnological targets [2, 6]. An example of this type of approach is the case of the CP12 protein, which modulates the assimilation of inorganic carbon by interacting with key enzymes of the Calvin-Benson cycle. CP12-deficient mutants have shown enhanced production of compounds of interest in different cyanobacteria [7–9].

Another interesting protein is CfrA (carbon-flow-regulator A), also known as PirC (PII-interacting regulator of carbon metabolism), a highly conserved regulator in cyanobacteria, that play a key role in adapting carbon flow in response to nitrogen deficiency [10, 11]. CfrA expression increases in *Synechocystis* during NtcA-dependent acclimation to nitrogen starvation [10, 12]. During this adaptive response (chlorosis), a large accumulation of glycogen takes place. CfrA promotes this accumulation by inhibiting 3-phosphoglycerate mutase (PGAM), enzyme that directs fixed carbon towards the lower glycolysis pathway by converting 3-phosphoglycerate into 2-phosphoglycerate. CfrA also interacts with the regulatory protein PII depending on the concentration of 2-oxoglutarate. When this metabolite increases due to its limited combination with nitrogen, PII releases CfrA favouring its interaction with PGAM and resulting in enhanced C flux towards glycogen [11]. The overexpression of *cfrA* triggers glycogen accumulation even in the presence of nitrogen and regardless of the presence of PII. Conversely, strains lacking CfrA exhibit greater carbon flow towards the lower glycolysis pathway and the TCA cycle for nitrogen combination and proteins synthesis [10]. Consistent with these findings, CfrA-deficient strains have been used to enhance the production of polyhydroxybutyrate (PHB) and ethanol, both products derived from pyruvate [13, 14]. A comparative analysis of *cfrA* overexpression was carried out in *Synechocystis* wild-type strains and those unable to store glycogen (Δ*glgC* genetic background), in both cases without eliminating the nitrogen source from the medium. In wild -type strains, CfrA accumulation leads to the derivation of C towards glycogen and other compounds such as sucrose and glucosylglycerol (GG), whereas in the Δ*glgC* background, the C is reallocated towards different Calvin-Benson cycle intermediates and amino acids [15]. These data highlight the biotechnological potential of modulating *cfrA* expression to redirect carbon flux for sugar production.

Microbial cell factories based on heterotrophic organisms such as *Escherichia coli* or *Saccharomyces cerevisiae* require an abundant and sustainable supply of sugar feedstocks to be viable for technological applications. Therefore, engineering cyanobacteria for the photosynthetic production of sugars could be a promising alternative. Sucrose production has been previously investigated in *Synechocystis* and other cyanobacteria, where it functions as an osmolyte [5, 16–18]. Another well-established role of sucrose is as a fixed carbon carrier molecule in filamentous cyanobacteria, transporting it between the vegetative cells where it is produced and the heterocysts where it is utilized [19]. Glycogen is the main carbon storage compound in cyanobacteria, and its metabolism contributes in an essential way to the metabolic plasticity required for growth in diurnal cycles and survival under different stress conditions [20, 21]. Although glycogen metabolism functions as the natural carbon sink, the synthesis of sucrose and GG ensures flexibility in the carbon sink network of cyanobacteria [17].

Numerous metabolic engineering strategies have been carried out for the photosynthetic production of sucrose in cyanobacteria, trying to bypass the natural regulatory mechanisms that control its synthesis and limit its accumulation. A key step is the use of transporters capable of secreting sucrose into the medium. The *E. coli cscB* gene was first used for this purpose in *Synechococcus elongatus* PCC 7942 [22] and has since been used with different expression approaches in other cyanobacteria. Additional modifications have included enhancing enzymes in the sucrose synthesis pathway and reducing its degradation [17, 18]. In other cases, pathways that can compete with sucrose, such as the synthesis of the osmoprotectant GG, have been eliminated, resulting in increased sucrose production [23, 24]. Various approaches have also targeted glycogen metabolism to improve the photosynthetic production of different products [25]. In the case of sucrose, the results have been variable and only in some cases have these strategies had a positive effect [26–28]. On the other hand, disturbing glycogen metabolism has a significant impact on the physiological robustness of cyanobacteria, so the use of these modifications should be carefully evaluated [17]. Another field of study that has received great interest in recent years is the co-cultivation of sucrose-secreting cyanobacteria with heterotrophic organisms, capable of using it as a carbon source and producing valuable compounds [29–33].

In this work, we focused on analysing the impact of CfrA regulator expression on sucrose production in *Synechocystis* and its close relationship with glycogen metabolism. We hypothesize that the inducible expression of CfrA could increase the yield of sucrose by boosting glycogen accumulation, which could then be used in the synthesis of this sugar. Our results demonstrate a clear positive effect of CfrA-mediated carbon partitioning on sucrose accumulation. Induction of *cfrA* expression in *Synechocystis* strains carrying a controlled expression system for a heterologous sucrose transporter (CscB from *E. coli*) and sucrose-phosphate synthase (SPS) (CscB/SPS system) improves sucrose productivity by 40% compared to control strains under the same conditions. Glycogen accumulation linked to CfrA expression contributes to enhanced sucrose synthesis. The presence of this carbon sink limits biomass generation and favours photosynthetic activity. Maximum sucrose production is achieved in a mutant lacking the synthesis of the osmolyte GG (Δ*ggpS*) cultivated in saline medium. In this strain, the expression of CfrA together with the CscB/SPS system results in the highest sucrose productivity.

## Methods

### Strain and culture conditions

To generate the P_*ars*_*-cfrA*/suc strain the autonomously replicating pDF-lac2-cscB-sps-CmR plasmid, containing a *cscB* gene codon-optimized for *Synechocystis* [24] was introduced into the previously described P_*ars*_*-cfrA* strain [10] by conjugation [34]. The strain Δ*ggpS*/P_*ars*_*-cfrA*/suc was generated by transformation of the P_*ars*_*-cfrA*/suc strain with the pSPARK-II-ΔggpS plasmid. To generate this plasmid the flanking regions of *sll1566* (GgpS CDS) were combined by overlapping PCR using *Synechocystis* genomic DNA and the oligonucleotides 5’AAGTCAAAAAGACTGGAAAATGGG3’ and 5’GATCGCggatccATCAGCGGTCTCCAAAATC3’ for the up region and 5’GCTGATggatccGCGATCGCCAATGCCAG3’and 5’CTTAACATCCTATGGGAAGGG3’ for the down region. This fragment containing a *Bam*HI restriction site was cloned into pSPARK-II (Canvax) and an Ery^r^ CCE1 cassette from pBR325 [34] was cloned in the *Bam*HI site. DNA constructs and strains segregation were verified by DNA sequencing and PCR analysis (Additional file 1 and 2: Fig. S1). A description of the strains and plasmids used in this work as well as the identification of the *Synechocystis* genes under study are shown in Table 1.

**Table 1:** *Synechocystis* strains, expression plasmid and genes identification

Name	Description	Expression plasmid	Gene ID
P_*ars*_-*cfrA*/suc	Δ*cfrA::aadA*^+^,*nrsD*::P_*arsB*_-*cfrA:npt* Sm/Sp^r^, Km^r^	pDF-lac2-cscB-sps-Cm^r^ [36]	*cfrA*- *sll0944* *ggpS*- *sll1556* *sps*- *sll0045*
Δ*ggpS*/P_*ars*_-*cfrA*/suc	Δ*ggpS::ermC*,Δ*cfrA::aadA*^+^,*nrsD*::P_*arsB*_-*cfrA:npt* Ery^r^,Sm/Sp^r^,Km^r^	pDF-lac2-cscB-sps-Cm^r^ [36]	

The proteins selected for overexpression were **CfrA** (carbon flow regulator A from *Synechocystis*), **CscB** (sucrose permease from *E. coli*) and **SPS** (sucrose-phosphate synthase from *Synechocystis*). **GGPS** (glucosylglycerol-phosphate synthase from *Synechocystis*) was targeted for inactivation. The antibiotic resistance markers used were **Sm/Sp**^**r**^ (streptomycin/spectinomycin), **Km**^**r**^ (kanamycin), **Ery**^**r**^ (erythromycin), and **Cm**^**r**^ (chloramphenicol).

*Synechocystis* sp. PCC 6803 derivative strains were grown photoautotrophically at 30 ºC on BG11 medium [35], supplemented with 1 g l^−1^ NaHCO_3_ (BG11C) and bubbled with 1% (v/v) CO_2_ in air, under continuous illumination (125 µmol of photons m^−2^ s^−1^; 4000 K LED lights), hereafter standard conditions. In some experiments this CO_2_ supplement was suppressed (air) or increased to 3% (v/v). In these experiments, BG11 buffered with 20 mM TES (N-[tris(hydroxymethyl)methyl]-2-aminoethanesulfonic acid) buffer pH 7.5 was used. In other cases, the analyses were carried out under different lighting conditions (200, 300 or 500 µmol of photons m^−2^ s^−^1). For plate cultures 1% (w/v) Bacto agar (Difco) and the required antibiotics were added (50 µg ml^−1^ kanamycin, 2.5 µg ml^−1^ spectinomycin, 2.5 µg ml^−1^ streptomycin, 10 µg ml^−1^ erythromycin and 10 µg ml^−1^ chloramphenicol). Precultures of the different strains from plate cultures were performed in 250 ml Erlenmeyer flasks containing 100 ml BG11C medium supplemented with 10 µg ml^−1^ chloramphenicol to maintain the presence of pDF-lac2-cscB-sps-CmR plasmid. This concentration of chloramphenicol was present in all cultures and should be sufficient to select for the presence of the plasmid during the experiment. Precultures were used to inoculate 150 ml cultures under standard conditions at 0.4 OD_750_. When these cultures reached approximately 0.8–1 OD_750_ the sucrose secretion experiments started by adding simultaneously IPTG (1 mM), NaCl and sodium arsenite (NaAsO_2_) at the different concentrations required in each experiment. Growth was monitored via optical density at 750 nm (OD_750_).

### Extraction and quantification of chlorophyll

Chlorophyll concentration from liquid cultures was determined by measuring the OD_665_ in methanolic extracts following Mackinney's protocol [36]. In alginate beads, chlorophyll extraction was carried out by adding 1 mL of methanol to samples containing 10 beads. The samples were incubated in the dark for one week with alternating periods of vortexing. Mackinney's protocol was then applied as to the rest of samples.

### Glycogen content determination

Glycogen was determined as previously described [15]. Standard curves of known amounts of amyloglucosidase-digested glycogen were employed for quantification.

### Sucrose content determination

Samples were taken from the cultures under different conditions. They were centrifuged and the supernatants stored at − 20 °C until analysis. Sucrose concentration was measured using the Sucrose/D-Glucose kit from Megazyme, according to the instructions of the manufacturer. Since bubbling differentially affects the evaporation of the cultures, to make the measurements comparable, the concentration of sucrose in each case was relative to the volume of the culture remaining at the time of each determination.

The maximum production rate was calculated from the maximum observed increase in sucrose concentration between two consecutive measurements (usually between day 1–2 or 2–3) in the different experiments. Maximum production in each case was calculated homogeneously on the sixth day after simultaneous induction of CfrA and the sucrose production/secretion system. In all conditions, at least two independent experiments (biological replicates) and three technical replicates of each measurement were carried out. The mean of the technical replicates and, in turn, the mean value of the biological replicates were calculated. These results were used to calculate the percentage increase when comparing two conditions or two strains. The data used in the calculations are shown in Table S1.

### Glucosylglycerol content determination

Frozen pellets from culture aliquots equivalent to 5 U OD_750nm_ were resuspended and incubated in 1 ml 80% ethanol for 4 h at 65 °C and the supernatants were recovered by centrifugation (5 min at 15,000 × *g*, RT) and dried in a vacuum centrifuge. Pellets were solubilized in 150 μL of 50% ultrapure water/methanol and filtered through 0.20 μm filters (Millex-GN Nylon, Millipore). Five μl samples were analysed by LC–MS. Chromatographic separation was performed with an XSELECT HSS XP 150 mm × 2.5 µm column (Waters) in an Exion HPLC (Sciex) connected to a Qtrap 6500 (Sciex) operating in negative mode. Sample data were acquired and processed with Analyst and SciexOs software. For quantification of the total amount of GG a calibration curve was performed with known quantities of this compound. The quantification was carried out in the chromatography service of the Instituto de Bioquímica Vegetal y Fotosíntesis (IBVF).

### Biomass quantification

The biomass produced in each condition was determined by quantifying the dry weight. For this purpose, 2–10 ml of culture, depending on the growth stage, were filtered using Mixed Cellulose Ester filters (0.45 µm). The filters were dried at 80 ºC overnight and the weight increase was calculated in each case.

### Photosynthetic measurements

Oxygen evolution was determined on cell cultures using a Clark-type electrode Chlorolab 2 + System; Hansatech) at 30 °C with continuous stirring. Cells were harvested at different times and adjusted to OD_750_ = 1. To prevent carbon limitation, cultures were supplemented with 10 mM NaHCO_3_ just before measurements. For O_2_ evolution at light saturation curves, light was provided by a LED light source (LED1, Hansatech).

Chlorophyll fluorescence measurements were performed with a DUAL-PAM-100 (Walz) using intact cells at room temperature. Before measurements, cells suspensions (5 µg of chlorophyll) were dark-adapted for 10 min. Effective quantum yield of PSII, Y(II), was calculated.

### Photosynthetic pigment analysis

Absorption spectra (350–750 nm) of *Synechocystis* cultures were measured. All spectra were normalised to 1 OD_750_ for the comparison of different samples.

### Immobilization of cells in alginate beads

Immobilization was carried out following previously described protocols with minor modifications [29, 31]. P_*ars*_*-cfrA*/suc cells grown in flasks with BG11C medium were collected by centrifugation when the culture reached 1.5 OD_750_ and resuspended in 1/24 of the original volume using 20 mM HEPES–NaOH, pH 7.5. The resulting suspension was homogeneously mixed with a 3% (w/v) alginate solution in a ratio of 1:12. The alginate-cell mixture was passed through a 25G hypodermic needle (0.5 × 16 mm) using a Pharmacia Biotech model P-1 peristaltic pump at a flow rate of 10 mL/min. The droplets from the pump fell into 50 mL Falcon tubes containing 40 mL of a 50 mM CaCl_2_ solution. Approximately 10 mL of the cell-alginate mixture was added to each tube. The droplets solidified immediately, forming beads approximately 2.5 mm in size. After 10 min, the CaCl_2_ solution was discarded, and the beads were washed twice with Milli-Q water. Finally, the beads were cultured under standard conditions in flasks containing 20 mL of beads and 80 mL of BG11C medium, bubbling with air enriched with 1% (v/v) CO_2_.

### Quantification of intracellular metabolites

For intracellular metabolite quantification, cells corresponding to 1–3 U OD_750_ were quickly collected by centrifugation (15 000 × *g* for 30 s at 4 °C) and immediately frozen in liquid N_2_ for storage at -80ºC until use. Metabolite extraction from frozen pellets was carried out as previously described [21]. For LC–MS analysis chromatographic separation was performed with an XSELECT HSS XP 150 mm × 2.5 µm column (Waters) in an Exion HPLC (Sciex) connected to a Qtrap 6500 (Sciex) operating in negative mode. Sample data were acquired and processed with Analyst and SciexOs software. For quantification of the total amount of the metabolites, different known concentrations of each standard were used. The quantification of metabolites was carried out in the chromatography service of the Instituto de Bioquímica Vegetal y Fotosíntesis (IBVF).

### Invertase enzymatic assay

Cells from 20 ml cultures in each condition analysed were harvested and resuspended in 250 μl of 20 mM Hepes–NaOH buffer (pH 7.0), 4 mM MgCl_2._ The cOmplete ULTRA tablets of Protease Inhibitor Cocktail from ROCHE were used. Crude extracts were prepared by mechanical disruption using glass beads in a FastPrep-24 5G; MP Biomedicals (at 6 m/s for 30 s). After centrifugation (15,000 × g 20 min at 4 °C), the soluble fraction was recovered. Protein concentration in cell-free extracts was determined by the method of Bradford, using ovalbumin as a standard [37]. The invertase activity was determined in 50 μl reaction mixture containing 20 mM Hepes–NaOH buffer (pH 7.0) and 100 mM sucrose. Reaction mixtures were incubated at 30 °C for 90 min and subsequently inactivated by incubation at 95 °C for 5 min. The quantification of glucose produced in the different reactions was carried out by LC–MS. Chromatographic separation was performed with an XSELECT HSS XP 150 mm × 2.5 µm column (Waters) in an Exion HPLC (Sciex) connected to a Qtrap 6500 (Sciex) operating in negative mode. Sample data were acquired and processed with Analyst and SciexOs software. For quantification of the total amount of glucose a calibration curve was performed with known quantities of this compound. The quantification was carried out in the chromatography service of the Instituto de Bioquímica Vegetal y Fotosíntesis (IBVF).

## Results

### Engineering *Synechocystis* sp. PCC 6803 to produce sucrose in strains with regulated CfrA expression.

**Fig. 1:**
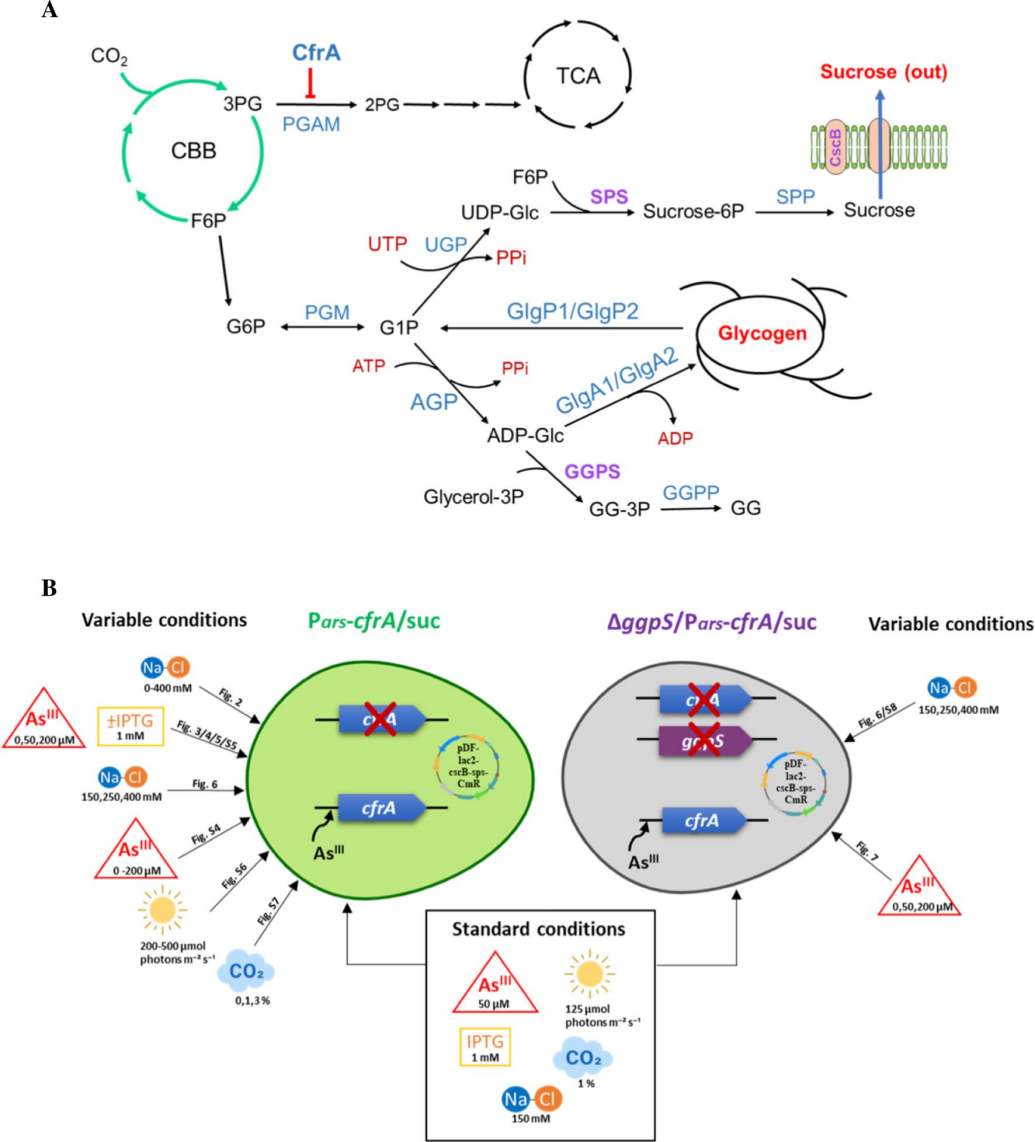
Schematic representation of carbon metabolism, genetic modifications and conditions analysed using *Synechocystis* sp. PCC 6803. **A** central carbon metabolism and carbohydrate biosynthetic pathways. Enzymes involved in the synthesis of glycogen, sucrose and glucosylglycerol (GG) as well as the enzyme inhibited by CfrA (PGAM) are shown in blue. The proteins engineered in this work (SPS, the permease CscB and GGPS) are highlighted in purple. **AGP**, ADP-glucose pyrophosphorylase; **CBB**, Calvin-Benson cycle; **CfrA**, carbon flux regulator A; **CscB,** sucrose permease; **GGPP**, glucosylglycerolphosphate phosphatase; **GGPS**, glucosylglycerolphosphate synthase; **GlgA1/GlgA2**, glycogen synthases; **GlgP1/GlgP2**, glycogen phosphorylases; **PGAM**, phosphoglycerate mutase; **PGM**, phosphoglucomutase; **SPS**, sucrose-phosphate synthase; **SPP**, sucrose-phosphate phosphatase; **TCA**, tricarboxylic acid cycle; **UGP**, UDP-glucose pyrophosphorylase. **B** schematic representation of the genetic modifications presents in the strains used in this work, as well as the conditions applied in the different experiments. Standard conditions are common to both strains and represent the fixed reference values ​​of each parameter versus the variable conditions used in the different experiments. We have chosen the color green for strain P_*ars*_-*cfrA*/suc and purple for strain Δ*ggpS*/P_*ars*_-*cfrA*/suc. In each figure, the different color intensities indicate the variable condition in each case

A simplified representation of carbon metabolism in *Synechocystis* and the main carbon sinks from glucose-1-phosphate intermediate is shown in Fig. 1A. The genetic modifications and metabolic conditions analysed throughout the work are outlined in Fig. 1B. In order to evaluate the possible role of the CfrA regulator in the sucrose production in *Synechocystis*, a strain with regulated CfrA expression, mediated by an arsenite-inducible promoter (P_*ars*_*-cfrA*) was engineered to increase sucrose biosynthesis and its secretion into the culture medium. The previously described autoreplicative plasmid pDF-lac2-cscB-sps-CmR [24] was introduced into the P_*ars*_*-cfrA* strain, giving rise to the P_*ars*_*-cfrA*/suc strain. This strain expresses *Synechocystis* sucrose-phosphate synthase (SPS) and *E. coli* sucrose transporter CscB under the control of an IPTG-inducible promoter. The induction of *sps* gene expression was verified by semiquantitative RT-PCR (Additional file 1and 3: Fig. S2) and overexpression of the SPS enzyme was observed in the protein profiles of the P_*ars*_*-cfrA*/suc strain (Additional file 1 and 4: Fig. S3). Previous results with the P_*ars*_*-cfrA* parental strain demonstrated that the addition of arsenite at the concentrations used in this work did not negatively affect its growth [15].

**Fig. 2:**
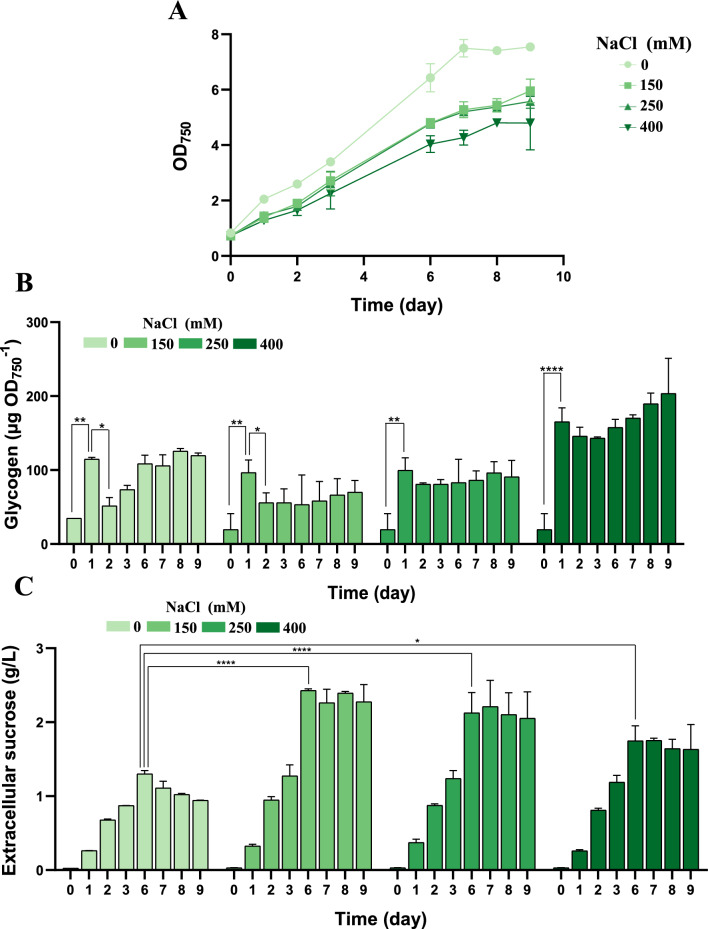
Influence of saline concentration on sucrose production in P_*ars*_*-cfrA*/suc strain. Samples were taken over 9 days, before (day 0) and after the addition of 50 µM arsenite, 1 mM IPTG and the indicated amounts of NaCl. **A** growth analysis, **B** glycogen content, and **C** extracellular sucrose accumulated in the culture supernatants. Error bars in (**A**), (**B**), and **C** represent SD of the mean values from 2 independent experiments (with 3 technical replicates). Asterisks indicate significant difference using ANOVA test (*P < 0.05, **P < 0.01, ***P < 0.001, and ****P < 0.0001)

Given the osmoprotective role of sucrose in *Synechocystis*, we analysed the production of this osmolyte in cultures of the P_*ars*_*-cfrA*/suc strain under standard conditions with different salt concentrations and arsenite as an inducer of *cfrA* expression. The NaCl concentrations tested were within a range that does not significantly affect growth, according to previous studies [16], and the arsenite concentration that led to the accumulation of a significant amount of CfrA in the parental P_*ars*_*-cfrA* strain (50 µM) [10]. A control without NaCl was included. Growth, extracellular sucrose and glycogen accumulation were monitored over time (Fig. 2). Growth was only slightly slowed in the presence of salt, consistent with the moderately halotolerant character of *Synechocystis* (Fig. 2A). Regarding glycogen accumulation, the induction of the sucrose production system (by IPTG addition) together with the expression of CfrA (by arsenite addition) caused a rapid increase in this polymer, a typical effect of CfrA [10], clearly observable 24 h after the start of the experiment at all salt concentrations (Fig. 2B). However, in the following hours glycogen level decreased temporarily and then increased progressively, to a greater extent at high salt concentrations. Simultaneously with the initial decrease in glycogen, the amount of sucrose secreted into the culture medium increased over time (Fig. 2C). The peak of sucrose accumulation occurred around the sixth day of culture, remaining without major variations until the end of the experiment. The amount of sucrose secreted was around 50% higher in the presence of salt, reaching a maximum in cultures with 150 mM NaCl. This salinity level was therefore used for subsequent studies.

### Partitioning of fixed carbon in the P_*ars*_*-cfrA*/suc strain after induction of the sucrose secretion system and effect on photosynthetic activity

**Fig. 3:**
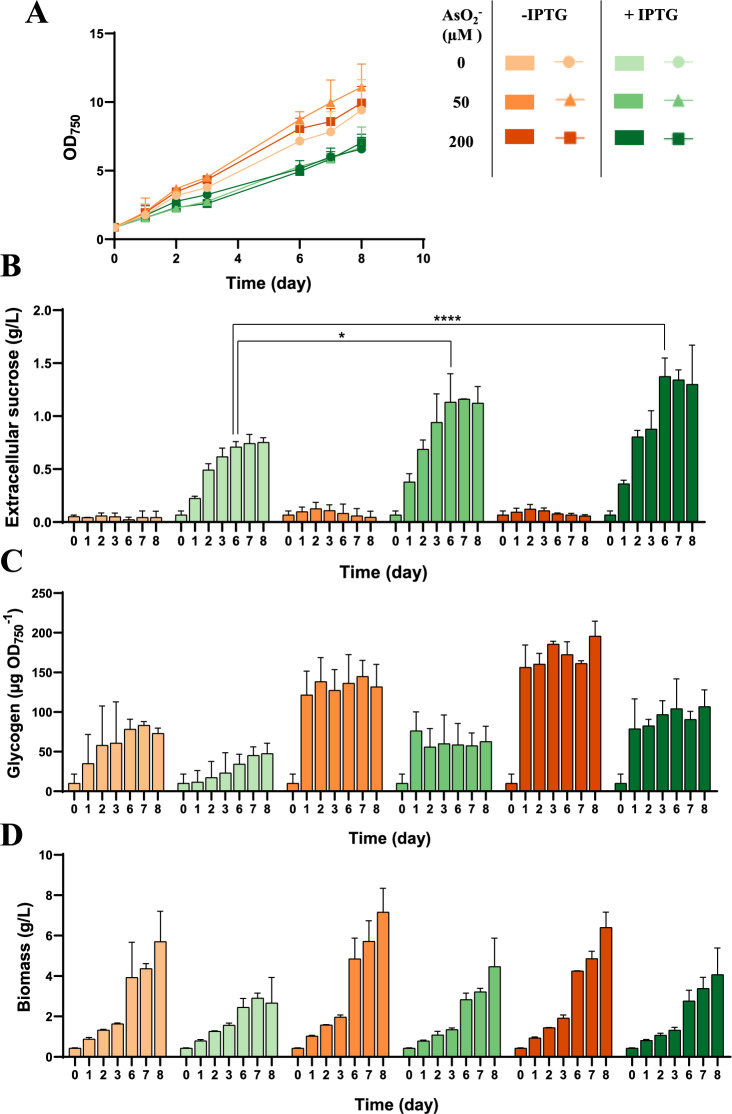
Effect of activating the sucrose production/secretion system in the P_*ars*_*-cfrA*/suc strain. Samples were collected over 8 days following the addition of different concentrations of AsO₂⁻ (0, 50, or 200 µM) and 150 mM NaCl in the presence or absence of 1 mM IPTG. A) growth analysis, **B** extracellular sucrose accumulated in the culture supernatants, (**C**) glycogen content, (**D**) carbon partitioning (biomass/glycogen/extracellular sucrose) in P_*ars*_*-cfrA*/suc strain. Error bars in (**A**), (**B**), (**C**) and (**D**) represent SD of the mean values from 2 independent experiments (with 3 technical replicates). Asterisks indicate significant difference using ANOVA test (*P < 0.05 and ****P < 0.0001)

Previous research demonstrated that increasing arsenite levels led to higher CfrA accumulation and a simultaneous rise in glycogen content in the P_*ars*_*-cfrA* parental strain [10]. To further explore how CfrA accumulation affects sucrose production and its connection to glycogen synthesis, the P_*ars*_*-cfrA*/suc strain was grown under standard conditions with 150 mM NaCl and varying arsenite concentrations (0–200 µM). The previously described effects associated with CfrA expression (decrease in photosynthetic pigments and glycogen accumulation) were monitored under these conditions. As expected, a clear positive correlation between added arsenite and glycogen accumulation was observed. Sucrose production was clearly enhanced in the presence of arsenite even at low concentrations of this CfrA inducer (Additional file 1: Fig. S4). To further characterize the influence of CfrA on the production of sucrose and glycogen, the effect of its accumulation in the cell was analysed independently or simultaneously with the expression of the sucrose synthesis system. For this purpose, the P_*ars*_*-cfrA*/suc strain was cultured under standard conditions, adding 150 mM of NaCl and increasing concentrations of arsenite (0, 50, 200 µM), both in the presence and absence of IPTG (1 mM). As shown in Fig. 3A, the addition of IPTG caused a delay in the growth of this strain, both with and without arsenite. The analysis of glycogen amount, as well as the sucrose secreted into the medium, again demonstrated the positive effect of CfrA on sucrose production in cultures induced with both arsenite and IPTG. As expected, minimal sucrose secretion was observed in cultures without IPTG, regardless of CfrA induction (Fig. 3B). The CfrA-mediated increase was around 40% compared to control cultures without arsenite, both in total productivity and maximum production rate (Table 2). However, increasing arsenite concentration had a limited effect on total sucrose production. These results could indicate a limitation in the sucrose production system, unlike what was observed for glycogen accumulation, when arsenite is increased.

**Table 2:** Effect of CfrA on maximum sucrose production (g/L) and maximum sucrose production rate (mg L^−1^ h^−1^)

Strain	CfrA	Maximum (g/L)	Increase (%)	Maximum productivity (mg L^−1^ h^−1^)	Increase (%)
P_*ars*_*-cfrA*/suc	– +	0.89 1.54	42	12.58 20.06	37
Δ*ggpS*/P_*ars*_*-cfrA*/suc	– +	1.68 2.72	36	20.29 38.64	47

The average of the maximum values reached in the experiments in which the induction ( +) or not ( − ) of CfrA has been compared is shown. Strains P_*ars*_*-cfrA*/suc and Δ*ggpS*/P_*ars*_*-cfrA*/suc were cultured under standard conditions (125 μmol photons m⁻^2^ s⁻^1^, 30 °C, 1% (v/v) CO_2_ in air). The maximuns of extracellular sucrose were reached with 150 mM NaCl and 200 μM arsenite in the case of the P_*ars*_*-cfrA*/suc strain and 400 mM NaCl and 50 μM arsenite for the Δ*ggpS*/P_*ars*_*-cfrA*/suc strain. In both cases the maximun was calculated on the sixth day of cultivation. Maximun productivity was calculated using the largest increase in total sucrose secreted between two consecutive measurements, in most cases between the first and second day of culture after the addition of inducers.

Glycogen accumulation increased as a function of CfrA levels and was much higher when the sucrose secretion system was not induced ( − IPTG) (Fig. 3C). Under these conditions, accumulated glycogen reached 40% of cell biomass, whereas under the same conditions but with IPTG, this percentage was around 20% (Additional file 1: Fig. S5A). The biomass produced, estimated by dry weight, was also higher in cultures untreated with IPTG, consistent with the observed OD_750_.

The analysis of the production/secretion of sucrose relative to the quantity of cells at each time, measured as g/L/OD_750_, shows, as expected, a greater production in the initial stages of the culture, decreasing progressively as the culture ages and light is limited by shading (Additional file 1: Fig. S5B).

We wanted to determine if the induction of *cfrA* and/or the sucrose production/secretion system by IPTG had any influence on invertase activity levels and, therefore, on the amount of sucrose hydrolyzed under different conditions. As shown in Additional file 1: Figure S5C, invertase activity did not change with the addition of IPTG and around a 30% increase was observed after the addition of arsenite at the maximum concentration (200 μM).

Figure 3D shows a global analysis of carbon partitioning into biomass, glycogen, and sucrose under the different conditions. These results indicate that induction of the sucrose secretion system leads to a decrease in biomass and glycogen production, with a greater carbon flow directed toward sucrose production.

**Fig. 4:**
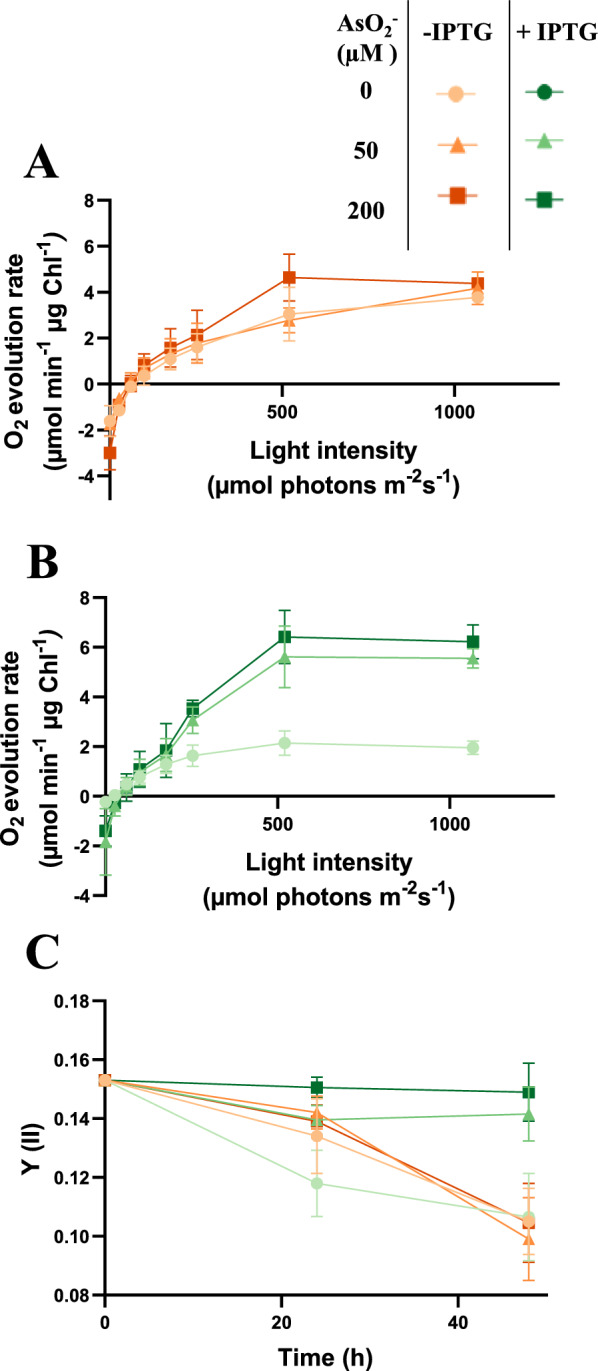
Effects of *cfrA* overexpression and sucrose production/secretion activation on the photosynthetic parameters of P_*ars*_*-cfrA*/suc strain. **A** and **B** light saturation curves of P_*ars*_*-cfrA*/suc strain cells normalized to chlorophyll content, measured 48 h after the addition of arsenite (0, 50, or 200 μM), 150 mM NaCl and without IPTG (panel A) or with IPTG (panel B). **C** operating efficiency of PSII [Y(II)] in the P_*ars*_*-cfrA*/suc strain under growth light conditions (125 µmol photons m⁻^2^ s⁻^1^) before (0 h) and after (24 and 48 h) arsenite (0, 50, or 200 μM), NaCl and IPTG (if applicable) addition. Error bars in (**A**), (**B**) and (**C**) represent SD of the mean values from 2 independent experiments (with 3 technical replicates)

To determine whether sucrose secretion as a carbon sink influences photosynthetic activity, as previously described [38–40], the oxygen evolution rate was evaluated in P_*ars*_*-cfrA*/suc strain cells treated or not with IPTG, under different arsenite concentrations. Measurements were taken 48 h after the addition of IPTG at different light intensities and normalized to the chlorophyll content of each sample. In IPTG treated strains, the addition of arsenite resulted in light saturation curves with peak values up to three times higher than those observed in cells not exposed to arsenite (Fig. 4B). However, the curves were very similar and with clearly lower maxima in cells not treated with IPTG, regardless of adding arsenite (Fig. 4A). In line with these findings, pulse amplitude modulation fluorometry revealed that the operational efficiency of photosystem II [Y(II)] was highest in cells treated with both IPTG and arsenite after 48 h, surpassing all other samples (Fig. 4C). These results indicate a positive effect on the photosynthetic activity when sucrose production is enhanced by CfrA expression.

The contribution of increased light intensity and CO_2_ input to carbon partitioning in the P_*ars*_*-cfrA*/suc strain was also analysed. To evaluate the effect of increasing light intensity on sucrose production, the P_*ars*_*-cfrA*/suc strain was cultured under standard conditions with 150 mM NaCl, 50 μM arsenite, and 1 mM IPTG, while varying the light intensity (200, 300, or 500 μmol photons m⁻^2^ s⁻^1^). Growth was not compromised under any of the conditions studied. Accumulated glycogen was significantly higher in cultures illuminated with 500 μmol photons m⁻^2^ s^−1^. Compared to standard conditions of 125 μmol photons m⁻^2^ s⁻^1^ light intensity, sucrose secretion was slightly increased at higher irradiances (200, 300, or 500 μmol photons m⁻^2^ s⁻^1^), reaching maxima around 2–2.3 g/L, but with no significant differences in production among these conditions (Additional file 1: Fig. S6).

To analyse the effect of carbon supply, the P_*ars*_*-cfrA*/suc strain was grown under standard conditions, in the presence of 150 mM NaCl, 50 μM arsenite and 1 mM IPTG, but using BG11 medium. Cultures were bubbled with air (0.04% CO_2_) or air supplemented with 1% (v/v) or 3% (v/v) CO_2_. Limiting CO_2_ availability resulted in a severe growth delay. Despite the ability to store carbon in glycogen, sucrose accumulation was negligible under these conditions. On the other hand, increasing CO_2_ input to 3% did not result in higher sucrose secretion than in cultures with 1% CO_2_. This finding indicates that carbon allocation towards sucrose secretion requires facilitated CO_2_ fixation, which is achieved with a 1% CO_2_ supply (Additional file 1: Fig. S7).

### Metabolic profile of the P_*ars*_*-cfrA*/suc strain based on the expression of the sucrose production/secretion system

**Fig. 5:**
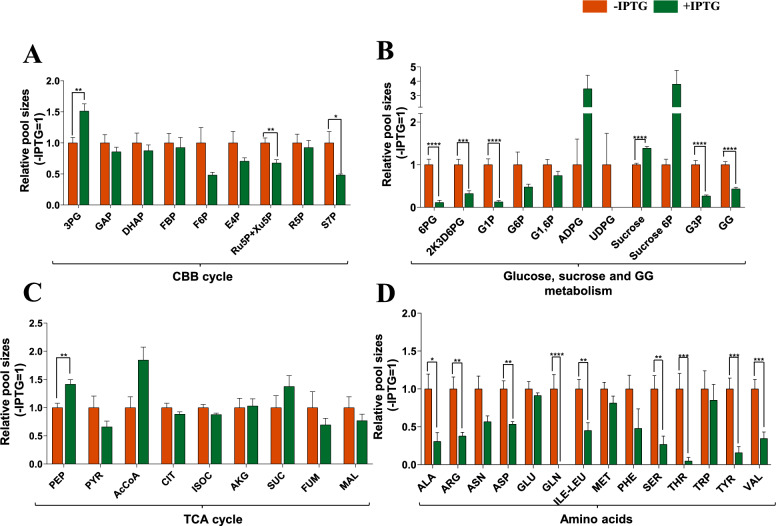
Intracellular metabolite pool sizes at 48 h post induction in P_*ars*_*-cfrA*/suc strain. Samples were collected 48 h after the addition of 150 mM NaCl and 50 µM arsenite in the presence (green) or absence (orange) of 1 mM IPTG. Data represent the means ± SEM of at least five biological replicates. Asterisks indicate significant difference using Student’s *t* test (*P < 0.05, **P < 0.01, ***P < 0.001, and ****P < 0.0001)

In order to analyse the metabolic effects of sucrose production, we performed a targeted metabolomics study of the P_*ars*_*-cfrA*/suc strain, either induced or not for the expression of the sucrose production/secretion system. Two cultures were grown under standard conditions. Once they reached an OD_750_ of approximately 0.8–1, 150 mM NaCl and 50 µM arsenite were added to both cultures, and IPTG was added to only one. After 48 h of treatment, samples were taken from both cultures (± IPTG) for metabolomic analysis. The sizes of the intracellular metabolite pools under sucrose secretion conditions relative to the control without IPTG are shown in Fig. 5. In general, the abundance of Calvin-Benson cycle intermediates decreased in the IPTG-induced culture compared to the control culture. However, the pool of 3PG, the initial product of carbon fixation and the metabolite representing the branching point between the Calvin-Benson cycle and the lower glycolytic pathway, increased when the sucrose production and secretion system (+ IPTG) was active (Fig. 5A). Glucose-6P-derived metabolites experienced a significant decrease when the sucrose sink was induced, either those from its oxidation by alternative routes such as 6-phosphogluconate or the intermediate 2-keto-3-deoxy-6-phosphogluconate or from its conversion to the precursor of glycogen and sucrose, glucose-1-phosphate. As expected, the pools of sucrose synthesis substrates, Fructose-6P and UDP-glucose, were also lower under IPTG induction conditions. Greater accumulation of intracellular sucrose was observed in IPTG-induced cultures (Fig. 5B). However, this accumulation also occurred in uninduced controls, as previously described in CfrA overexpression studies [15]. Salt stress was present in the cultures analysed here, which undoubtedly contributed to the accumulation of sucrose via the SPS enzyme. However, sucrose secretion into the medium was negligible in uninduced cultures (Fig. 3B), due to the IPTG-dependent expression of the CscB transporter. In general, total amino acid pool decreased in the sucrose-secreting cultures, indicating a reduced combination of carbon and nitrogen into biomass under conditions of sucrose sink induction (Fig. 5D). The TCA cycle intermediates did not experience major changes when inducing the sucrose production system, although some metabolites from the lower glycolytic pathway (PEP, Acetyl-CoA) increased slightly in the induced cultures (Fig. 5C).

### Sucrose secretion in the P_*ars*_*-cfrA*/suc strain with a Δ*ggpS* genetic background, unable to synthesize GG.

**Fig. 6:**
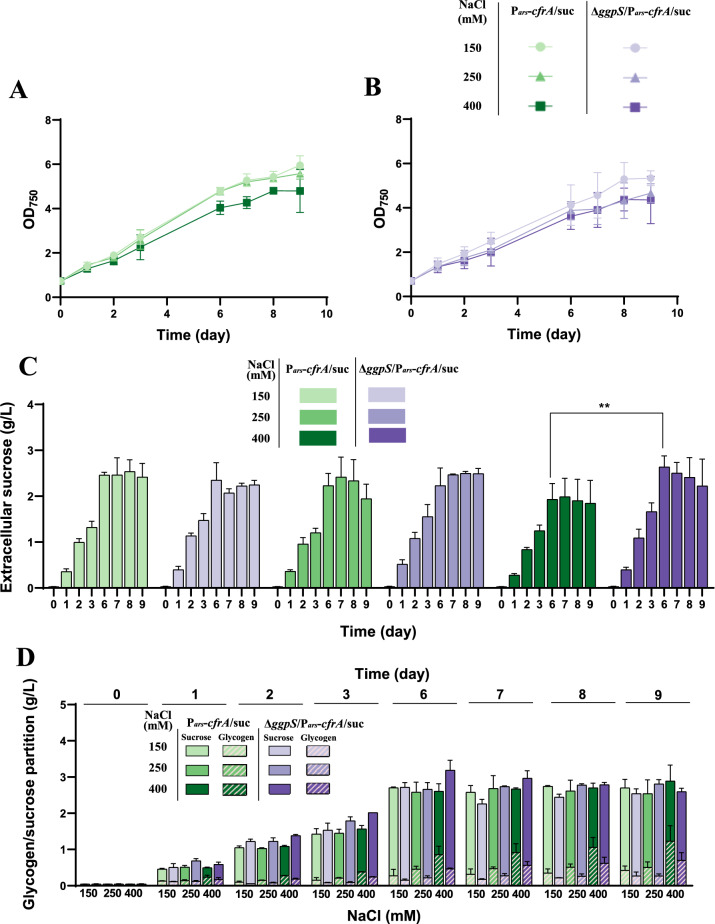
Influence of saline concentration on sucrose production/secretion in P_*ars*_*-cfrA*/suc and Δ*ggpS*/P_*ars*_*-cfrA*/suc strains. Samples were collected over a 9-day period, before (day 0) and after the addition of 50 µM arsenite, 1 mM IPTG and NaCl at the indicated concentrations (150, 250, or 400 mM). **A** and **B** growth analysis of P_*ars*_*-cfrA*/suc and Δ*ggpS*/P_*ars*_*-cfrA*/suc strains, respectively. **C** extracellular sucrose accumulated in the culture supernatants of P_*ars*_*-cfrA*/suc and Δ*ggpS*/P_*ars*_*-cfrA*/suc strains. **D** carbon partitioning (glycogen/extracellular sucrose) in both strains. Error bars in (**A**), (**B**), (**C**) and **D** represent SD of the mean values from 2 independent experiments (with 3 technical replicates). Asterisks indicate significant difference using ANOVA test (**P < 0.01)

As mentioned in the introduction, *Synechocystis* accumulates both sucrose and GG in response to salt stress. Previous work has shown that removing the GG synthesis pathway can enhance sucrose production [16, 24]. To test this approach in the P_*ars*_*-cfrA*/suc strain, we generated a variant that lacked the *ggpS* gene, encoding glucosylglycerol phosphate synthase (Δ*ggpS*/P_*ars*_*-cfrA*/suc). Since GG as an osmolyte accumulates in greater quantities at high saline concentrations in a wild-type *Synechocystis* strain [21], we cultured the P_*ars*_*-cfrA*/suc and Δ*ggpS*/P_*ars*_*-cfrA*/suc strains under standard conditions, adding 50 μM arsenite and different NaCl concentrations (150, 250 and 400 mM). The P_*ars*_*-cfrA*/suc strain showed only a slight reduction in growth at the highest salt concentration (400 mM), whereas the growth of the Δ*ggpS*/P_*ars*_*-cfrA*/suc strain was slightly affected at both 250 and 400 mM NaCl (Fig. 6A and B). Both strains exhibited the same pattern of carbon distribution observed in previous analyses, although glycogen accumulation was lower at all salt concentrations in the Δ*ggpS*/P_*ars*_*-cfrA*/suc strain compared to the P_*ars*_*-cfrA*/suc strain (Additional file 1: Fig. S8A). At the highest salt concentration (400 mM), this difference was more pronounced, and the decrease in glycogen was accompanied by increased sucrose production. Under these conditions, maximum sucrose production was around 30% higher in the Δ*ggpS*/P_*ars*_*-cfrA*/suc strain than in the P_*ars*_*-cfrA*/suc strain (Fig. 6C), reaching the maximum sucrose content per liter obtained in this study (2.72 g/L) (Table 2). Partitioning between glycogen and sucrose in both strains under the different conditions is shown in Fig. 6D. Glycogen content increases significantly with rising salt concentration, regardless of the *ggpS* gene mutation (Additional file 1: Fig. S8A).

To complete the study of the partitioning of carbon flow among the three compounds that share the precursor glucose-1-phosphate (glycogen, sucrose, and GG), we analysed the accumulation of GG in the P_*ars*_*-cfrA*/suc and Δ*ggpS*/P_*ars*_*-cfrA*/suc strains under different salt conditions. As shown in Additional file 1: Fig. S8B, the addition of 150–250 mM NaCl causes an increase in GG accumulation in the P_*ars*_*-cfrA*/suc strain; however, greater salt stress (400 mM) does not lead to greater GG synthesis. Interestingly, under these latter conditions, the cells accumulate a significantly higher amount of glycogen (Additional file 1: Fig. S8A). As expected, no detectable amounts of GG accumulated in the Δ*ggpS*/P_*ars*_*-cfrA*/suc strain.

**Fig. 7:**
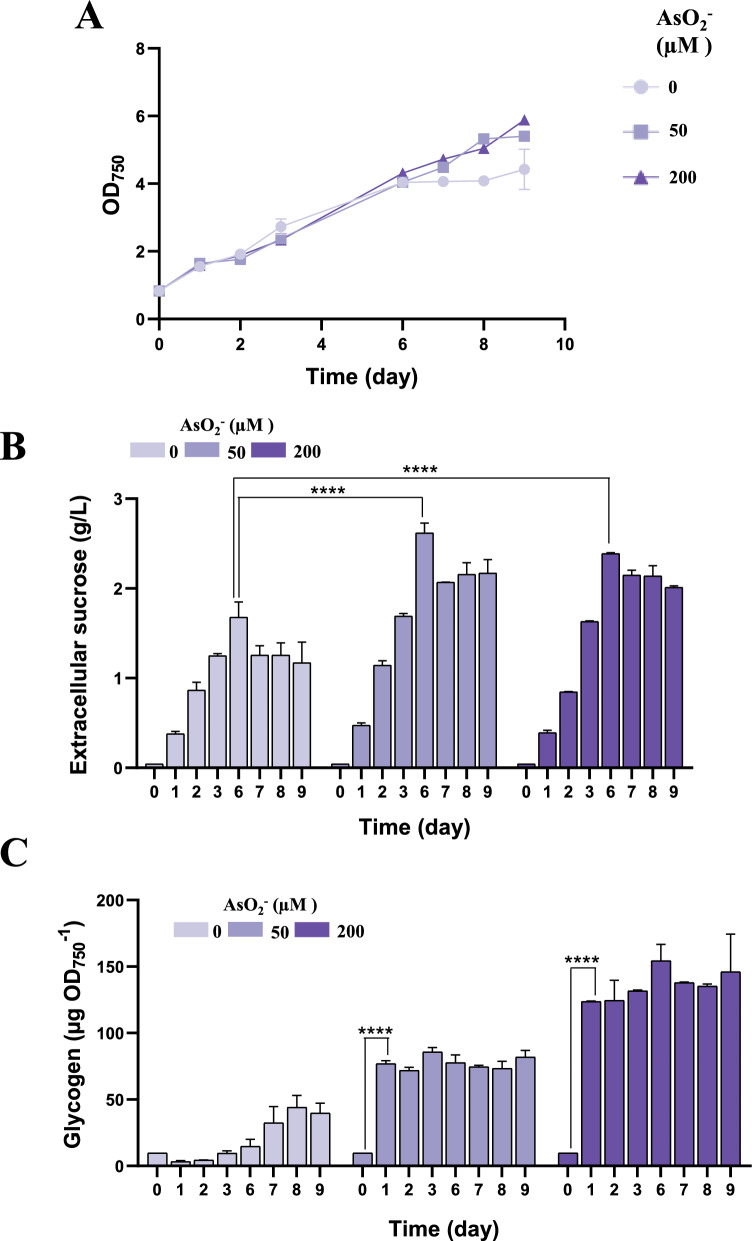
Effect of *cfrA* overexpression on sucrose production/secretion in Δ*ggpS*/P_*ars*_*-cfrA*/suc strain during high salinity stress. Samples were collected over a 9 days period, before (day 0) and after the addition of 400 mM NaCl, 1 mM IPTG and different concentrations of arsenite (0, 50, or 200 µM). **A** growth analysis, **B** glycogen content and **C** extracellular sucrose accumulated in the culture supernatants. Error bars in (**A**), (**B**), and (**C**) represent SD of the mean values from 2 independent experiments (with 3 technical replicates). Asterisks indicate significant difference using ANOVA test (****P < 0.0001)

Considering the observed positive effect of the *ggpS* mutation on sucrose production, we analysed whether the Δ*ggpS*/P_*ars*_*-cfrA*/suc strain could further increase sucrose production by raising CfrA induction levels. The Δ*ggpS*/P_*ars*_*-cfrA*/suc strain was cultured under standard conditions with 400 mM NaCl and increasing concentrations of arsenite (0, 50 and 200 µM). Growth was not affected by increasing arsenite concentration (Fig. 7A). CfrA induction with 50 µM arsenite increased sucrose production by 47% (Table 2). However, there was no greater increase with higher arsenite concentrations (Fig. 7B). Nevertheless, the increase in CfrA was reflected in glycogen accumulation levels (Fig. 7C). This behaviour is analogous to that observed in the P_*ars*_*-cfrA*/suc strain depending on the arsenite concentration (Fig. 3).

### Sucrose production in encapsulated P_*ars*_*-cfrA*/suc strain cells

**Fig. 8:**
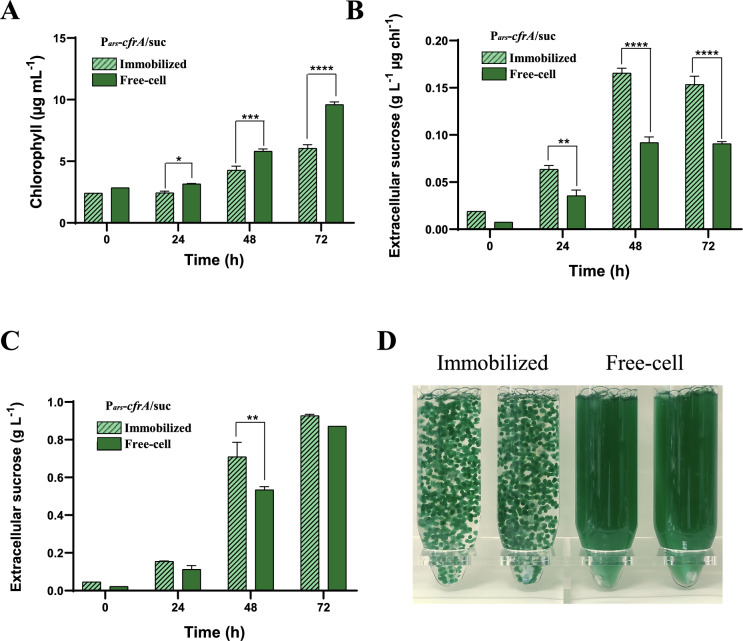
Effect of immobilizing P_*ars*_*-cfrA*/suc strain cells on sucrose production/secretion. **A** comparative growth analysis of cells in suspension and immobilized monitored via chlorophyll content, **B** extracellular sucrose accumulated in the culture supernatants of immobilized and free-cell P_*ars*_*-cfrA*/suc strain, related to the amount of chlorophyll, **C** absolute extracellular sucrose accumulated in the culture supernatants of immobilized and free-cell P_*ars*_*-cfrA*/suc strain. Samples were collected over a 72-h period, before (0 h) and after the addition of 150 mM NaCl, 50 µM arsenite and 1 mM IPTG. **D** Picture of P_*ars*_*-cfrA*/suc cells after 24 h of culture: immobilized (left) and free-cell (right). Error bars in (**A**), (**B**) and (**C**) represent SD of the mean values from 2 independent experiments (with 3 technical replicates). Asterisks indicate significant difference using ANOVA test (*P < 0.05, **P < 0.01, ***P < 0.001, and ****P < 0.0001)

One improvement strategy applied in sucrose production studies has been the immobilization of secretory strains in alginate [31]. Therefore, we investigated whether immobilizing the P_*ars*_*-cfrA*/suc strain in alginate beads could increase sucrose production (Fig. 8). In parallel, suspension cells of the same strain were cultured under standard conditions. Immobilization slowed growth, as monitored by chlorophyll accumulation. However, immobilized cells accumulated up to 25% more sucrose than free cells, 48 h after induction of the production system (0.71 g/L versus 0.53 g/L). This increase rises to 47% based on chlorophyll content. These results suggest the possibility of optimizing sucrose production in the P_*ars*_*-cfrA*/suc strain when immobilized in these structures. An image of the standard suspension and immobilized cultures is shown (Fig. 8D).

## Discussion

The model cyanobacterium *Synechocystis* was selected for this study for two main reasons. First, it is the organism in which the carbon flux regulator CfrA has been identified and studied [10]. Second, *Synechocystis* is a moderately halotolerant cyanobacterium that naturally synthesizes sucrose as a compatible osmolyte when exposed to salt stress [16]. This ability attenuates the requirement of freshwater for its cultivation. Sucrose synthesis is an early response to salinity increase in *Synechocystis*, although its accumulation is transient after a salt-shock, while GG accumulates progressively in these conditions. An ion-mediated reverse activation of the enzymes responsible for sucrose synthesis (SPS) and its degradation (Invertase) probably causes the accumulation pattern of this osmolyte in *Synechocystis*, depending on the acclimatization process to salt stress [21, 23]. In our sucrose-secreting strains, IPTG-dependent expression of the plasmid-encoded *sps* gene is added to the salt-regulated expression of the endogenous *sps* gene. However, *cscB* gene expression is exclusively IPTG-dependent. For this reason, extracellular sucrose accumulation is negligible in the absence of IPTG (Fig. 3), whereas intracellular sucrose accumulates to considerable levels even in the absence of IPTG, at least during the first 48 h (Fig. 5B). In these strains, sucrose secretion clearly increases in the presence of NaCl (Fig. 2C), indicating that there is a regulation of SPS expression and activity by salt stress. The P_*ars*_*-cfrA*/suc strain secreted the highest amounts of sucrose at moderate NaCl concentrations (150 mM) (Fig. 2C). However, at higher concentrations (400 mM), the Δ*ggpS*/P_*ars*_*-cfrA*/suc strain, which is unable to synthesize GG, secreted more sucrose (Fig. 6C), consistent with previous studies in *Synechocystis ggpS* mutants [16]. Similarly, a high and progressive accumulation of sucrose has been described in response to high salt concentrations (500 mM NaCl) for a Δ*glgC* mutant. This strain is also unable to synthesize GG because it does not synthesize ADP-glucose, a substrate of the GGPS enzyme, although it does not survive this salt stress [21]. In line with previous studies in other cyanobacteria [41], raising salt stress leads to slower growth and increased glycogen accumulation in both P_*ars*_*-cfrA*/suc and Δ*ggpS*/P_*ars*_*-cfrA*/suc strains (Fig. 2A and B; Fig. 6A, B, D).

Several studies have indicated a close relationship between intracellular pools of sucrose and glycogen. In *S. elongatus* PCC 7942, it has been shown that a progressive decrease in *glgC* expression limits rather than enhances sucrose production. Overexpression of *glgC* alone does not increase sucrose production, but it does so in combination with overexpression of the *sps* gene [27]. The effect of increasing glycogen degradation by regulating the enzyme GlgP on sucrose production has also been explored [28]. These findings suggest that glycogen may serve as a carbon reservoir for sucrose synthesis, rather than competing with it for carbon flow [27]. Chemical triggers of glycogen accumulation have been analysed in *Synechocystis*. The use of these chemicals could represent a complementary strategy for redirecting carbon flow toward glycogen synthesis and its subsequent utilization in sugar synthesis [42]. However, other studies in *Synechococcus* sp. PCC 7002 have shown that null mutants in glycogen synthases genes exhibit increased production of soluble sugars, such as sucrose [26]. The negative effect of the *glgC* mutation on sucrose synthesis could be due to the fact that the SPS enzyme might utilizes ADP-glucose in vivo, in addition to UDP-glucose, as has been described in vitro for the *Synechocystis* SPS [43]. Interestingly, the previously characterized P_*ars*_*-cfrA* strain showed sucrose accumulation upon CfrA induction in the absence of salt stress, whereas the Δ*glgC*/P_*ars*_*-cfrA* strain did not [15]. The strains analysed in this study consistently exhibited a sharp increase in glycogen accumulation dependent on the arsenite-mediated induction of CfrA. This effect was evident 24 h after simultaneous treatment with arsenite and NaCl. However, in the following hours, a decrease in the glycogen pool occurred, which was more pronounced in cultures with less arsenite and, consequently, less CfrA and glycogen (Additional file 1: Fig. S4). This decrease was not observed in cultures without IPTG (Fig. 3C). Together with others data showing glycogen mobilization in the wild-type *Synechocystis* strain under salt stress [21], these results clearly indicate that at least part of the glucose-1-phosphate required for osmolyte synthesis (Fig. 1) comes from glycogen degradation. In this sense, the metabolic effect of CfrA diverting the flow of carbon preferentially towards glycogen synthesis, as opposed to lower glycolysis and its combination with nitrogen, is of special relevance in sucrose production. In fact, a control of the flux towards lower glycolytic pathways and biomass synthesis has been suggested as a regulatory target of synthetic biology to enhance sucrose synthesis [41].

The biotechnological potential of metabolic engineering in cyanobacteria depends on an appropriate source-sink balance, which increases photosynthetic activity [40, 44]. Several studies have shown that the presence of an additional carbon sink positively impacts cellular physiology. It has been hypothesized that the additional energy consumption associated with this sink could release the full photosynthetic and metabolic potential of the cell, which would otherwise not operate at maximum capacity [40]. In our sucrose-secreting strains, establishment of a carbon sink, such as sucrose, was combined with CfrA-mediated regulation. CfrA induction increases the C/N ratio, due to a decreased nitrogen assimilation and redirection of carbon flow toward reserve compounds [15]. In this context, the functioning of the sucrose production system and its secretion into the medium led to a clear increase in photosynthetic activity (Fig. 4). Under these conditions, carbon partitioning was characterized by an increase in sucrose production to the detriment of biomass and glycogen accumulation in cultures treated with IPTG compared to untreated ones (Fig. 3D). Similar effects of salt stress on carbon allocation have been described in *S. elongatus* PCC 7942 modified for sucrose secretion [41]. In the case of the Δ*ggpS*/P_*ars*_*-cfrA*/suc strain, sucrose production was enhanced by the inability to synthesize GG. The glycogen content was lower than that of the P_*ars*_*-cfrA*/suc strain under the same conditions. The difference was more evident at higher saline concentrations (Fig. 6D; Additional file 1: Fig. S8A). This is probably because the synthesis of sucrose as an osmolyte is more relevant in a strain without GG, as has been described for a Δ*glgC* strain [21] and this synthesis takes place partly at the expense of glycogen. This inverse relationship between sucrose production and glycogen accumulation was also observed in cells of the P_*ars*_*-cfrA*/suc strain, whether or not subjected to salt stress (Fig. 2B and C) and has been previously reported in *S. elongatus* PCC 7942 [39].

The accumulation of GG in the P_*ars*_*-cfrA*/suc strain under salt stress (Additional file 1: Fig. S8B) is considerably lower than that reported for the wild-type strain under similar conditions [21]. This is particularly evident at high salt concentrations (400 mM). In accordance with this, the GG levels measured under standard conditions (150 mM NaCl, 50 μM arsenite) decrease significantly when the sucrose production system (+ IPTG) is activated (Fig. 5B). These data suggest that in a sucrose-producing strain, the synthesis of the osmolyte GG does not play a significant role in response to salt stress. Furthermore, in this strain, the combination of *cfrA* expression and the sucrose production system under high salt stress leads to carbon partitioning primarily towards glycogen synthesis.

A strategy aimed at increasing sucrose production by limiting biomass accumulation has been developed in *S. elongatus* PCC 7942. In this case, carbon redirection is carried out via an inducible growth arrest, through the constitutive expression of the regulator RpaB [45]. In a way, the effects mediated by the induction of CfrA in this study, decreasing nitrogen assimilation, lead to similar consequences on enhancing sucrose production. In both cases, these are inducible systems that can bypass genetic instabilities commonly associated with constitutive systems. Comparative metabolomic analysis of the P_*ars*_*-cfrA*/suc strain with and without IPTG revealed an increase in the first CO_2_ fixation product (3PG) under induction conditions (Fig. 5A), consistent with the increase in photosynthetic activity under these conditions (Fig. 4). However, the pool of most Calvin-Benson cycle intermediates is lower under conditions of sucrose secretion (Fig. 5A). This probably contributes to maintenance of high photosynthetic activity, by relieving feedback inhibition by sugar-phosphate intermediates when the sucrose sink is active (Fig. 4). The lower levels of glucose-1-phosphate and UDP-glucose, in the presence of IPTG, clearly point to their consumption as precursors for sucrose synthesis when this sink is induced. Considering these data, it might be thought that an increase in PGM or UGP activity could contribute to greater sucrose production (Fig. 1). Although some of these strategies have already been tested [16], we cannot rule out a possible positive effect in our conditions when CfrA is induced. The significantly lower accumulation of GG in cells treated with IPTG again demonstrated that the primary destination of glucose-1-phosphate is the synthesis of sucrose under these conditions. The overall decrease in amino acid pools in cells IPTG-treated is indicative of lower carbon–nitrogen combination for protein synthesis, which is consistent with the lower biomass production observed when the carbon flux to sucrose is high (Fig. 3).

In several of the conditions tested to enhance sucrose production in the P_*ars*_*-cfrA*/suc strain, a limitation was observed. This occurred when the amount of CfrA or light intensity increased, which positively affected glycogen accumulation but had little or no effect on sucrose production/secretion. This limitation could be due to the characteristics of the CscB permease or to limitations of the synthesis pathway itself under the conditions studied. Preliminary results from our laboratory demonstrate that this limitation could be overcome by replacing the culture medium when the sucrose concentration reaches its maximum, since the cells excrete sucrose again after this process. Similar strategies have been used in other studies [29]. In this sense, the use of sucrose produced by cyanobacteria, as a carbon source, by heterotrophic organisms that in turn synthesize high value compounds is a promising avenue, and can also contribute to keeping the sucrose production/secretion system active. Several approaches have been developed in this direction using different sucrose-secreting cyanobacteria [29, 32, 46, 47]. One of the fundamental challenges of these strategies is reconciling the growth conditions required by the species involved. It is possible that the generation of reactive oxygen species by photosynthetic organisms negatively affects the growth of heterotrophic species. In order to optimize the growth and productivity of these consortia, the immobilization of the cells, whether the phototrophic or heterotrophic partner, in hydrogel microstructures has been used successfully. [29–31]. In our system, the immobilization of cells of the P_*ars*_*-cfrA*/suc strain in alginate beads limits their growth and favours sucrose production at short times (Fig. 8). The fundamental advantage of this immobilization technique would be the easy recovery of the sucrose produced in these short-term cultures for use in different purposes, or the direct use of the culture medium for the cultivation of heterotrophic organisms of interest.

Regarding the use of the P_*arsB*_ promoter, it has been employed to establish the role of CfrA induction in sucrose production as a proof of concept, given its excellent on/off switching capability. However, other inducible promoters characterized in cyanobacteria could be used to avoid arsenite toxicity in biotechnological strategies.

The production of sucrose in cyanobacteria and its uses have recently been reviewed [18]. Most of the studies aimed at enhancing sucrose production/secretion have been carried out in *S. elongatus* PCC 7942, although also in other cyanobacteria including *Synechocystis*. These studies have shown that *Synechococcus elongatus* UTEX 2973, a fast-growing cyanobacterium, reaches the maximum production rate reported so far (8 g/L of extracellular sucrose) [48]. The results obtained in this study (Table 2) for the Δ*ggpS*/P_*ars*_*-cfrA*/suc strain are similar to those previously reported for a *Synechocystis* strain that is equally modified for deletion of the *ggpS* gene and that contains the same plasmid (pDF-lac2-cscB-sps-CmR) for expression of the *cscB* and *sps* genes [24]. However, induction of the *cfrA* gene in this strain significantly improves sucrose production, reaching a maximum production rate that is 47% higher than that obtained without inducing *cfrA* (Table 2).

## Conclusions

Accumulation of CfrA in the P_*ars*_*-cfrA*/suc strain increased maximum sucrose production by approximately 40%. In the Δ*ggpS*/P_*ars*_*-cfrA*/suc strain, the maximum increase was 47% compared to controls without CfrA. The maximum sucrose accumulation (2.72 g/L) was achieved in the Δ*ggpS*/P_*ars*_*-cfrA*/suc strain.

*cfrA* expression alongside the sucrose production/secretion system in the P_*ars*_*-cfrA*/suc strain resulted in a shift in carbon flow towards sucrose synthesis at the expense of biomass and glycogen. This contrasts with conditions where *cfrA* was expressed in the absence of the sucrose secretion system, which led to greater biomass generation and glycogen accumulation. The establishment of sucrose production as a carbon sink enhances the photosynthetic activity of the P_*ars*_*-cfrA*/suc strain, thereby increasing CO₂ fixation. As *cfrA* is a widely conserved gene among cyanobacteria [10, 11], whose expression can be finely regulated independently of nitrogen conditions [10], our results provide promising proof of concept regarding the role of this regulator in biotechnological approaches using different cyanobacteria.

## Supplementary Information

Additional file 1 (PPTX 4505 KB)

Additional file 2 (PPTX 52874 KB)

Additional file 3 (PPTX 52873 KB)

Additional file 4 (PPTX 435 KB)

## Data Availability

All data generated or analysed during this study are included in this published article [and its supplementary information files].
