# Supplementary material for: Enhanced sucrose production by controlling carbon flux through CfrA expression in Synechocystis sp. PCC 6803
Source: Microb Cell Fact. 2025 Dec 31;25:29. doi: 10.1186/s12934-025-02894-8 (PMC12853614; doi:10.1186/s12934-025-02894-8)

## Slide 1
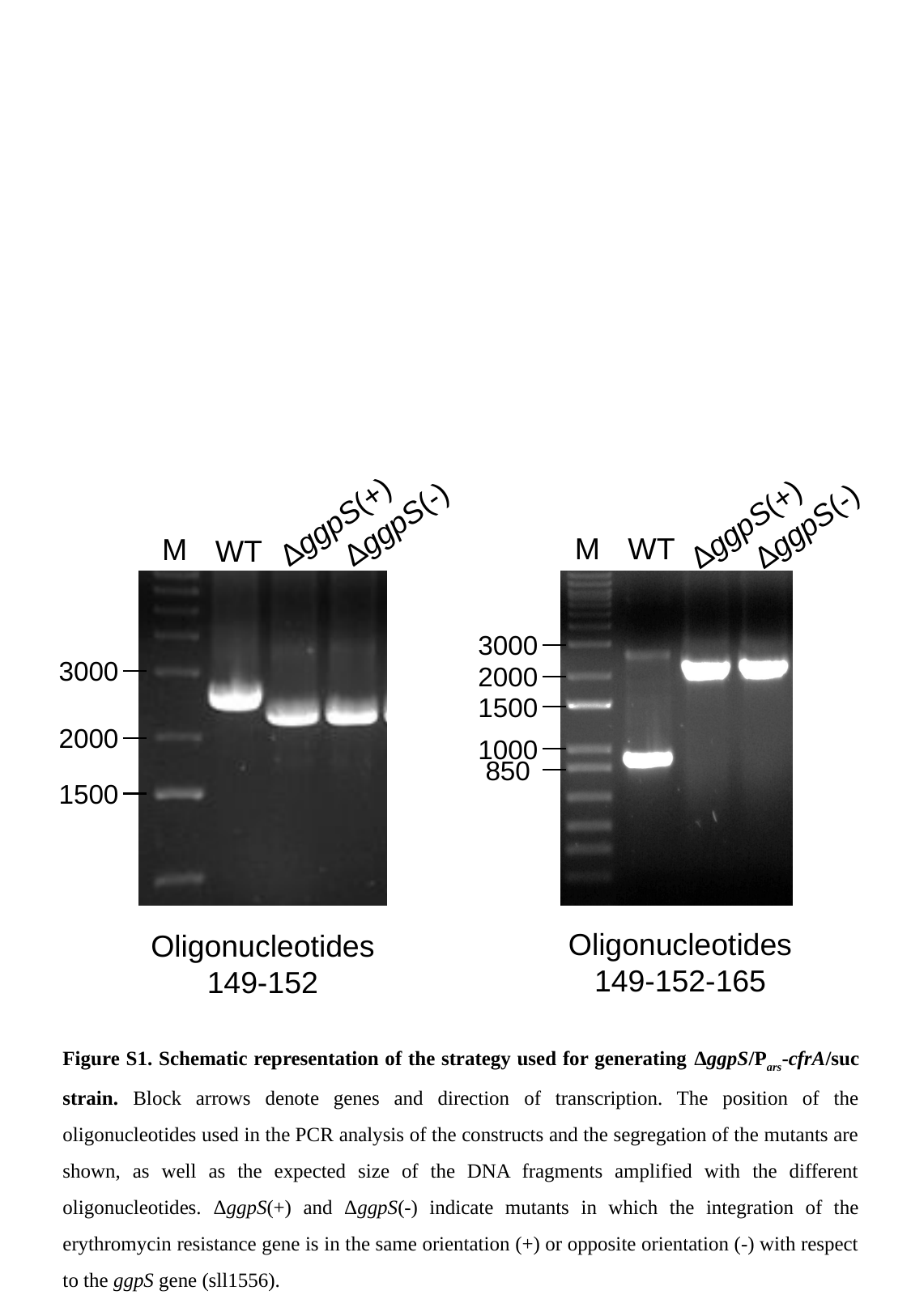

ΔggpS(+)
ΔggpS(-)
M
WT
3000
2000
1500
Oligonucleotides 149-152
ΔggpS(+)
ΔggpS(-)
WT
3000
2000
1500
1000
850
M
Oligonucleotides 149-152-165
Figure S1. Schematic representation of the strategy used for generating ΔggpS/Pars-cfrA/suc strain. Block arrows denote genes and direction of transcription. The position of the oligonucleotides used in the PCR analysis of the constructs and the segregation of the mutants are shown, as well as the expected size of the DNA fragments amplified with the different oligonucleotides. ΔggpS(+) and ΔggpS(-) indicate mutants in which the integration of the erythromycin resistance gene is in the same orientation (+) or opposite orientation (-) with respect to the ggpS gene (sll1556).

## Slide 2
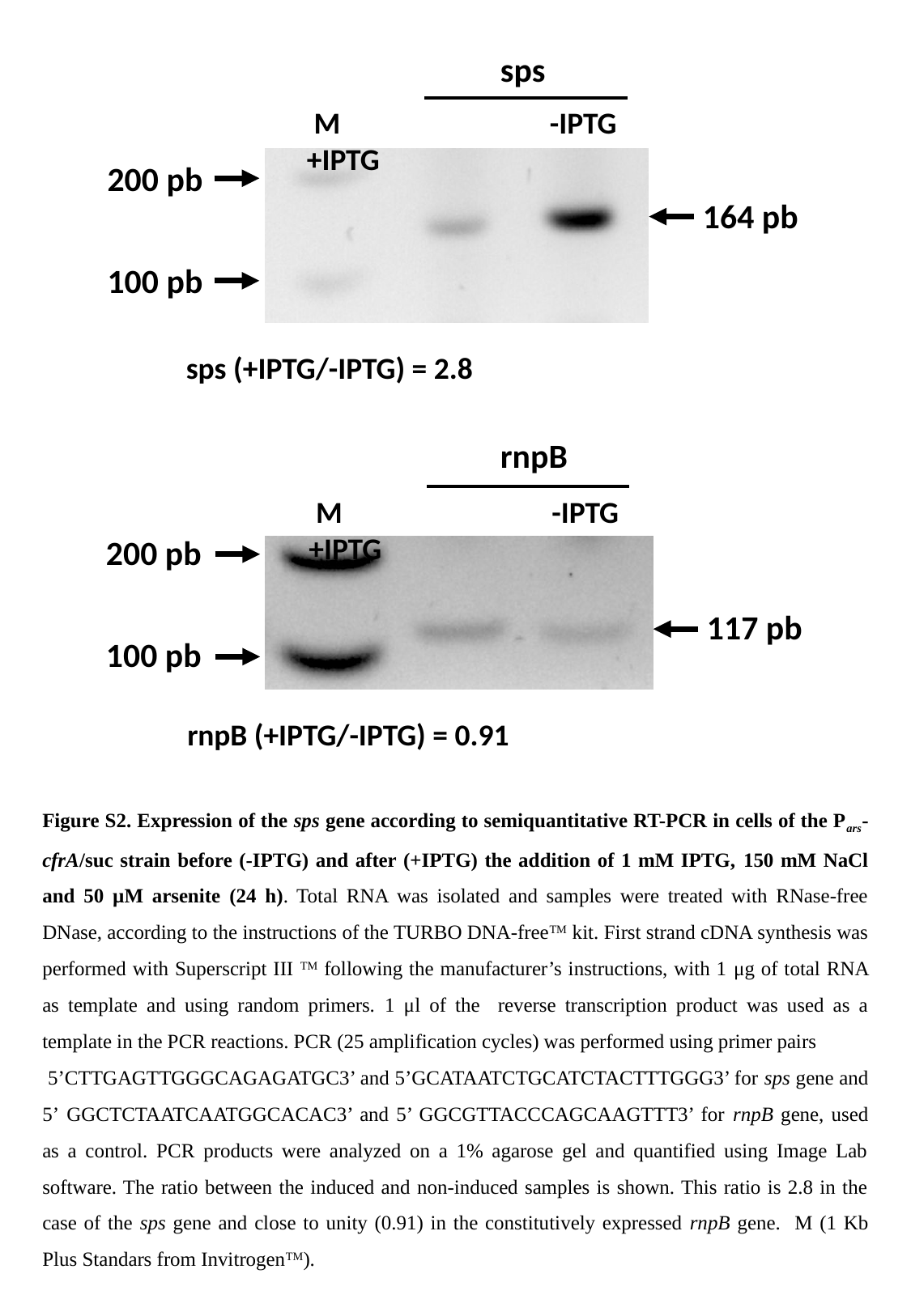

sps
 M		-IPTG	+IPTG
 200 pb
 164 pb
 100 pb
sps (+IPTG/-IPTG) = 2.8
 rnpB
 M		-IPTG	+IPTG
 200 pb
 117 pb
 100 pb
rnpB (+IPTG/-IPTG) = 0.91
Figure S2. Expression of the sps gene according to semiquantitative RT-PCR in cells of the Pars-cfrA/suc strain before (-IPTG) and after (+IPTG) the addition of 1 mM IPTG, 150 mM NaCl and 50 µM arsenite (24 h). Total RNA was isolated and samples were treated with RNase-free DNase, according to the instructions of the TURBO DNA-freeTM kit. First strand cDNA synthesis was performed with Superscript III TM following the manufacturer’s instructions, with 1 μg of total RNA as template and using random primers. 1 μl of the reverse transcription product was used as a template in the PCR reactions. PCR (25 amplification cycles) was performed using primer pairs
 5’CTTGAGTTGGGCAGAGATGC3’ and 5’GCATAATCTGCATCTACTTTGGG3’ for sps gene and 5’ GGCTCTAATCAATGGCACAC3’ and 5’ GGCGTTACCCAGCAAGTTT3’ for rnpB gene, used as a control. PCR products were analyzed on a 1% agarose gel and quantified using Image Lab software. The ratio between the induced and non-induced samples is shown. This ratio is 2.8 in the case of the sps gene and close to unity (0.91) in the constitutively expressed rnpB gene. M (1 Kb Plus Standars from InvitrogenTM).

## Slide 3
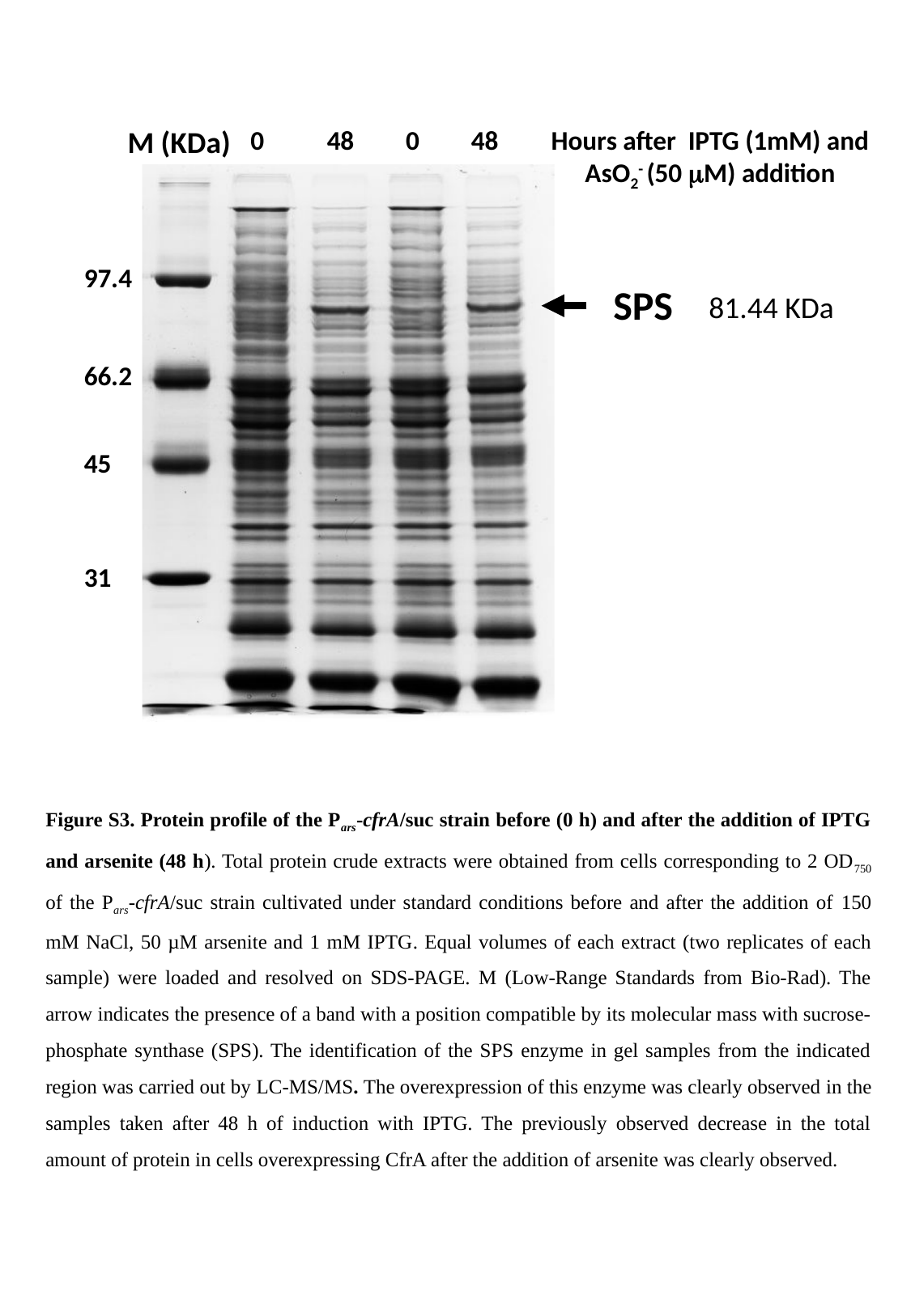

M (KDa)
0
48
0
48
Hours after IPTG (1mM) and AsO2- (50 mM) addition
97.4
SPS
81.44 KDa
66.2
45
31
Figure S3. Protein profile of the Pars-cfrA/suc strain before (0 h) and after the addition of IPTG and arsenite (48 h). Total protein crude extracts were obtained from cells corresponding to 2 OD750 of the Pars-cfrA/suc strain cultivated under standard conditions before and after the addition of 150 mM NaCl, 50 µM arsenite and 1 mM IPTG. Equal volumes of each extract (two replicates of each sample) were loaded and resolved on SDS-PAGE. M (Low-Range Standards from Bio-Rad). The arrow indicates the presence of a band with a position compatible by its molecular mass with sucrose-phosphate synthase (SPS). The identification of the SPS enzyme in gel samples from the indicated region was carried out by LC-MS/MS. The overexpression of this enzyme was clearly observed in the samples taken after 48 h of induction with IPTG. The previously observed decrease in the total amount of protein in cells overexpressing CfrA after the addition of arsenite was clearly observed.

## Slide 4
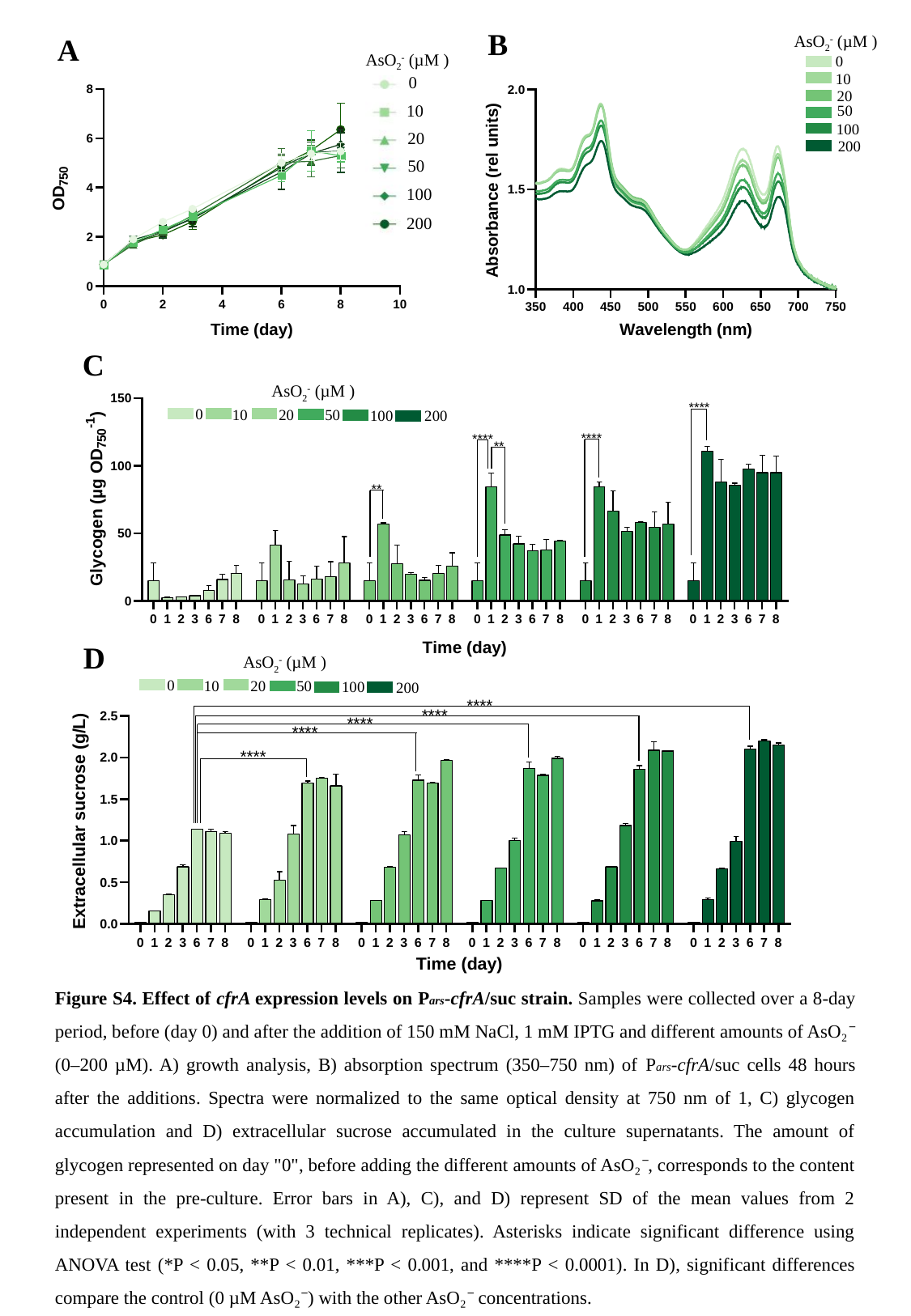

B
A
AsO2- (µM )
10
20
50
100
200
 0
AsO2- (µM )
0
10
20
50
100
200
C
AsO2- (µM )
0
10
20
50
100
200
****
****
| \*\*\*\* |
| --- |
**
| \*\* |
| --- |
D
AsO2- (µM )
0
10
20
50
100
200
| \*\*\*\* |
| --- |
| \*\*\*\* |
| --- |
| \*\*\*\* |
| --- |
| \*\*\*\* |
| --- |
| \*\*\*\* |
| --- |
Figure S4. Effect of cfrA expression levels on Pars-cfrA/suc strain. Samples were collected over a 8-day period, before (day 0) and after the addition of 150 mM NaCl, 1 mM IPTG and different amounts of AsO₂⁻ (0–200 µM). A) growth analysis, B) absorption spectrum (350–750 nm) of Pars-cfrA/suc cells 48 hours after the additions. Spectra were normalized to the same optical density at 750 nm of 1, C) glycogen accumulation and D) extracellular sucrose accumulated in the culture supernatants. The amount of glycogen represented on day "0", before adding the different amounts of AsO₂⁻, corresponds to the content present in the pre-culture. Error bars in A), C), and D) represent SD of the mean values from 2 independent experiments (with 3 technical replicates). Asterisks indicate significant difference using ANOVA test (*P < 0.05, **P < 0.01, ***P < 0.001, and ****P < 0.0001). In D), significant differences compare the control (0 µM AsO₂⁻) with the other AsO₂⁻ concentrations.

## Slide 5
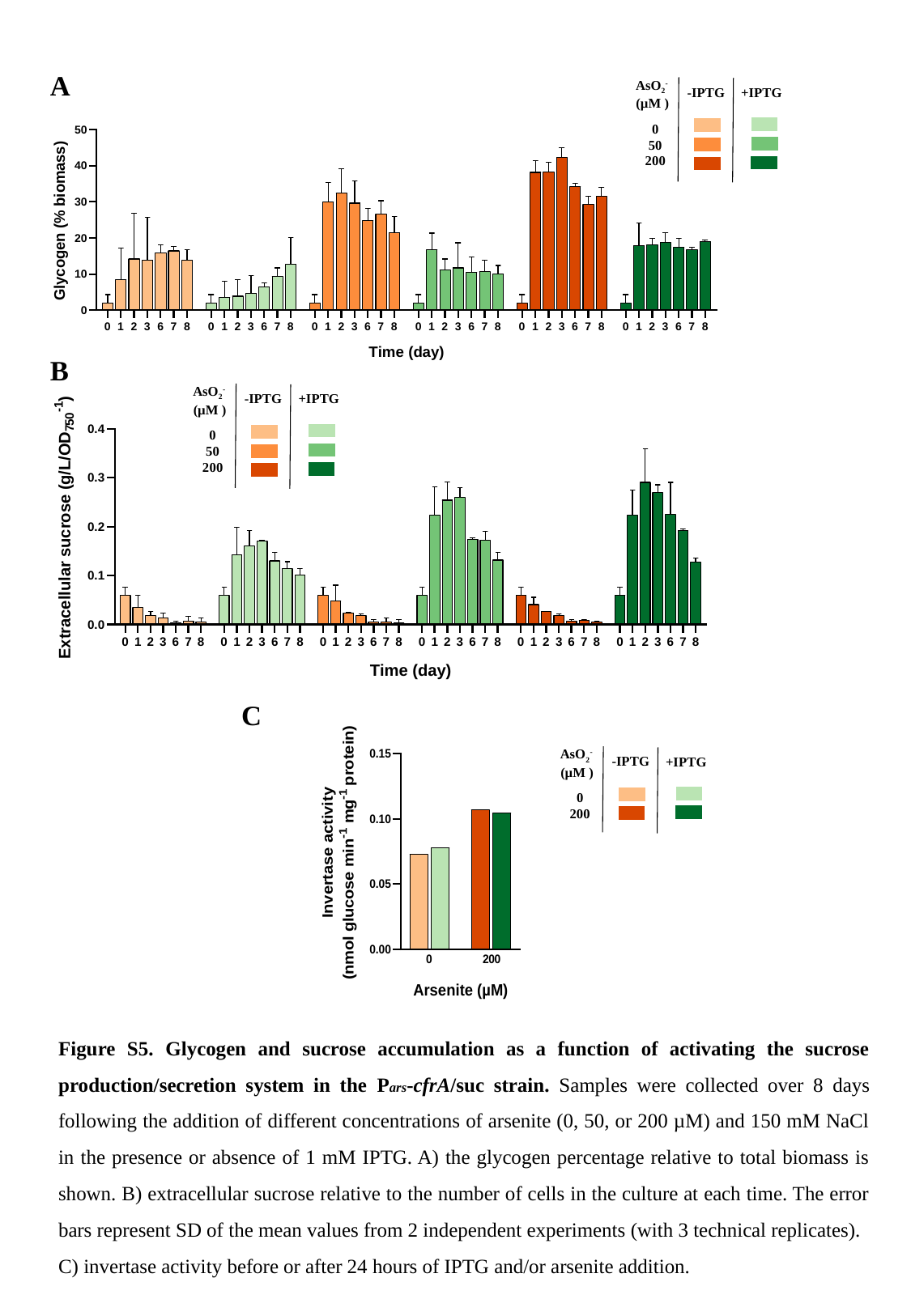

A
AsO2-
(µM )
0
50
200
 -IPTG
+IPTG
B
AsO2-
(µM )
0
50
200
 -IPTG
+IPTG
C
AsO2-
(µM )
0
200
 -IPTG
+IPTG
Figure S5. Glycogen and sucrose accumulation as a function of activating the sucrose production/secretion system in the Pars-cfrA/suc strain. Samples were collected over 8 days following the addition of different concentrations of arsenite (0, 50, or 200 µM) and 150 mM NaCl in the presence or absence of 1 mM IPTG. A) the glycogen percentage relative to total biomass is shown. B) extracellular sucrose relative to the number of cells in the culture at each time. The error bars represent SD of the mean values from 2 independent experiments (with 3 technical replicates).
C) invertase activity before or after 24 hours of IPTG and/or arsenite addition.

## Slide 6
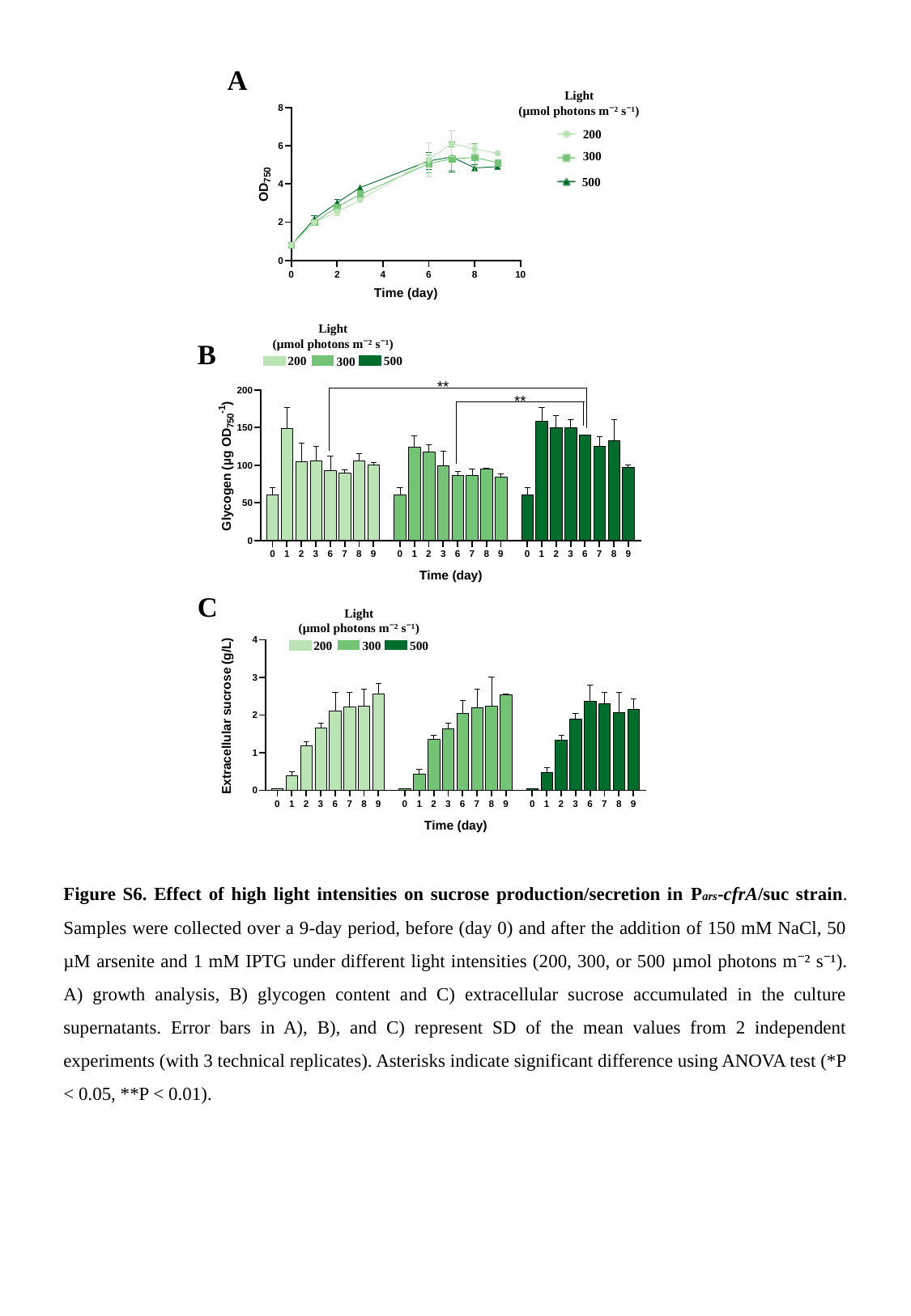

A
Light
(μmol photons m⁻² s⁻¹)
200
300
500
Light
(μmol photons m⁻² s⁻¹)
200
500
300
B
| \*\* |
| --- |
| \*\* |
| --- |
C
Light
(μmol photons m⁻² s⁻¹)
200
500
300
Figure S6. Effect of high light intensities on sucrose production/secretion in Pars-cfrA/suc strain. Samples were collected over a 9-day period, before (day 0) and after the addition of 150 mM NaCl, 50 µM arsenite and 1 mM IPTG under different light intensities (200, 300, or 500 µmol photons m⁻² s⁻¹). A) growth analysis, B) glycogen content and C) extracellular sucrose accumulated in the culture supernatants. Error bars in A), B), and C) represent SD of the mean values from 2 independent experiments (with 3 technical replicates). Asterisks indicate significant difference using ANOVA test (*P < 0.05, **P < 0.01).

## Slide 7
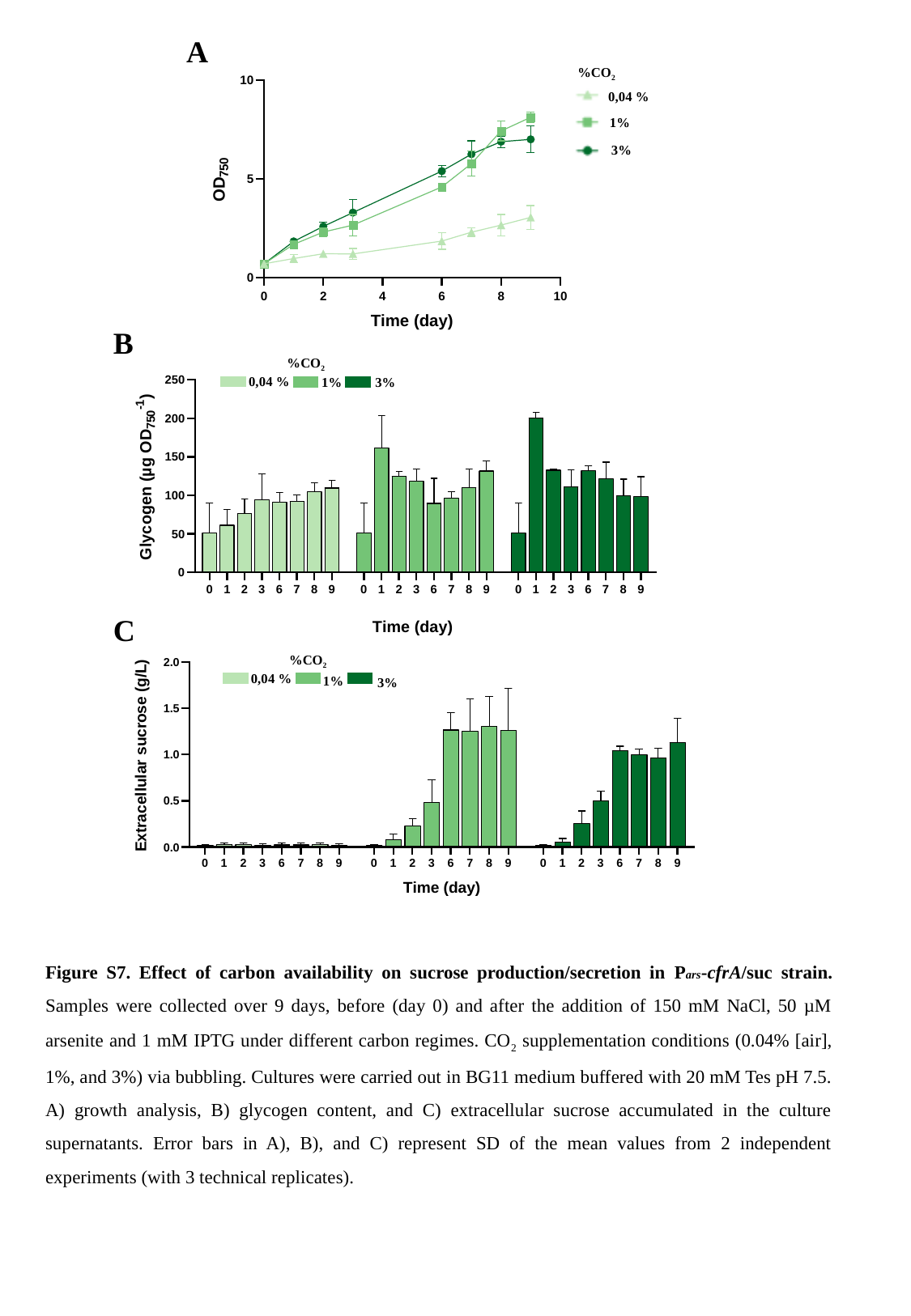

A
%CO2
0,04 %
1%
3%
B
%CO2
0,04 %
1%
3%
C
%CO2
0,04 %
1%
3%
Figure S7. Effect of carbon availability on sucrose production/secretion in Pars-cfrA/suc strain. Samples were collected over 9 days, before (day 0) and after the addition of 150 mM NaCl, 50 µM arsenite and 1 mM IPTG under different carbon regimes. CO2 supplementation conditions (0.04% [air], 1%, and 3%) via bubbling. Cultures were carried out in BG11 medium buffered with 20 mM Tes pH 7.5. A) growth analysis, B) glycogen content, and C) extracellular sucrose accumulated in the culture supernatants. Error bars in A), B), and C) represent SD of the mean values from 2 independent experiments (with 3 technical replicates).

## Slide 8
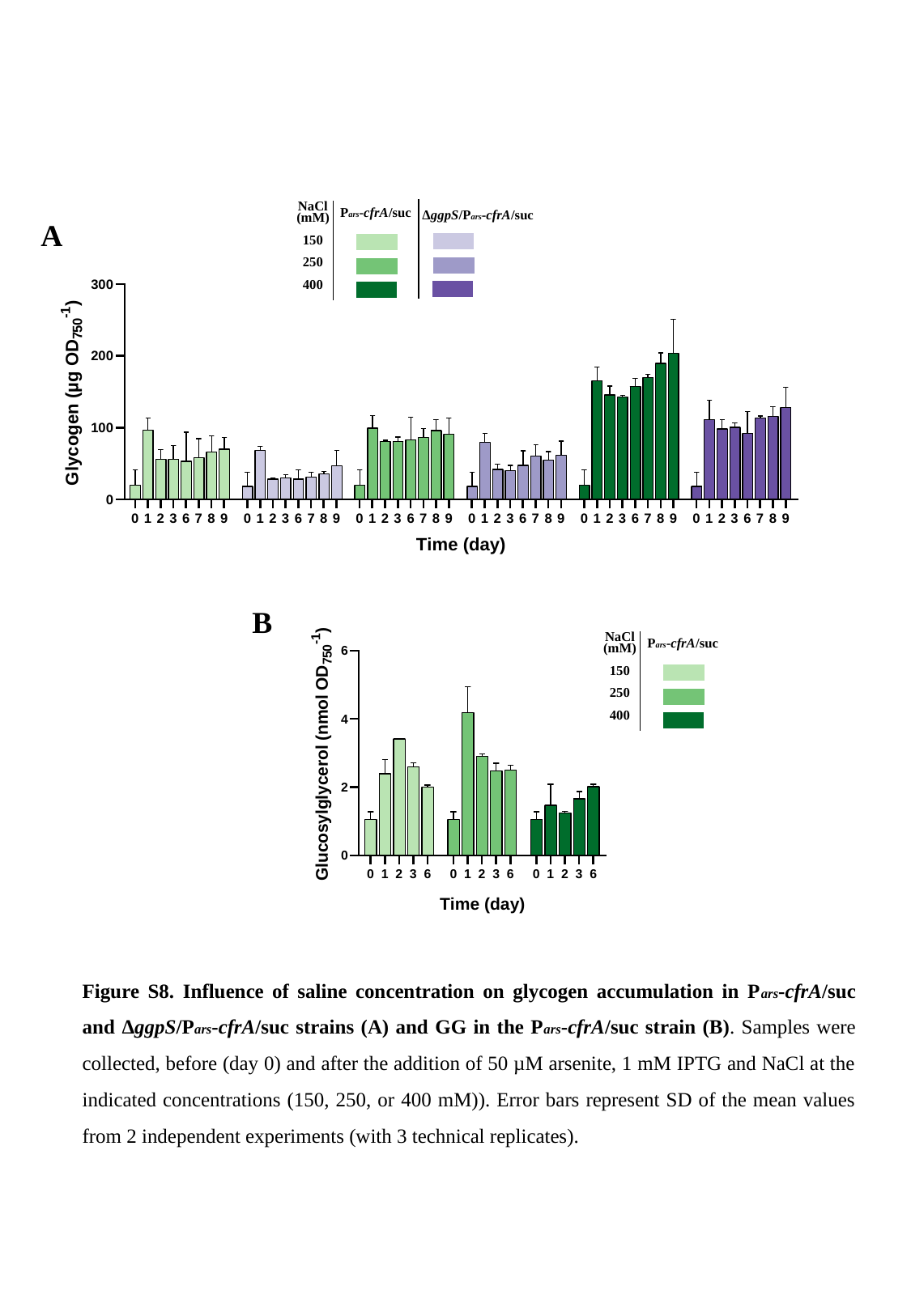

ΔggpS/Pars-cfrA/suc
NaCl (mM)
150
250
400
Pars-cfrA/suc
A
B
NaCl (mM)
150
250
400
Pars-cfrA/suc
Figure S8. Influence of saline concentration on glycogen accumulation in Pars-cfrA/suc and ΔggpS/Pars-cfrA/suc strains (A) and GG in the Pars-cfrA/suc strain (B). Samples were collected, before (day 0) and after the addition of 50 µM arsenite, 1 mM IPTG and NaCl at the indicated concentrations (150, 250, or 400 mM)). Error bars represent SD of the mean values from 2 independent experiments (with 3 technical replicates).

## Slide 9
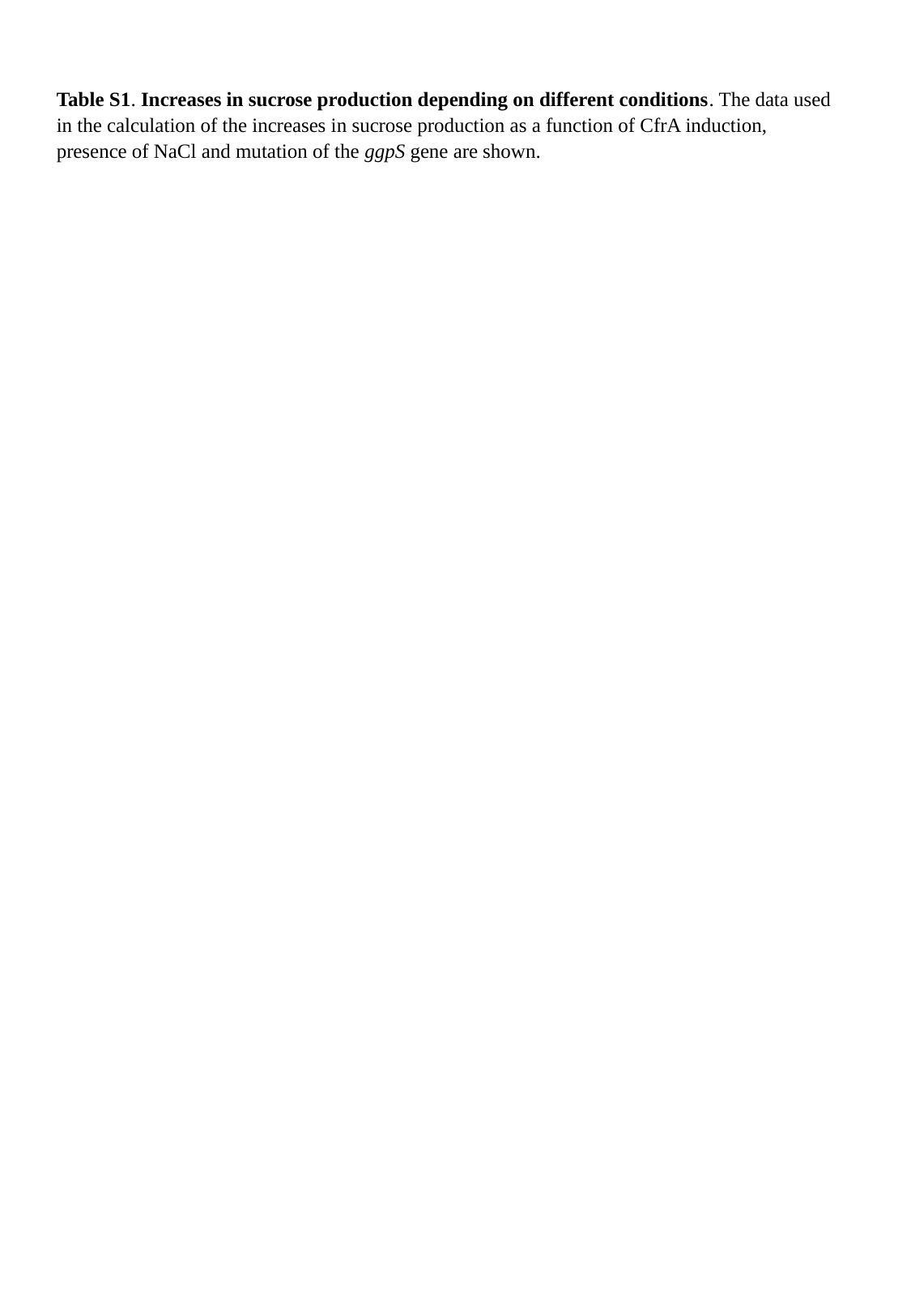

Table S1. Increases in sucrose production depending on different conditions. The data used in the calculation of the increases in sucrose production as a function of CfrA induction, presence of NaCl and mutation of the ggpS gene are shown.

## Slide 10
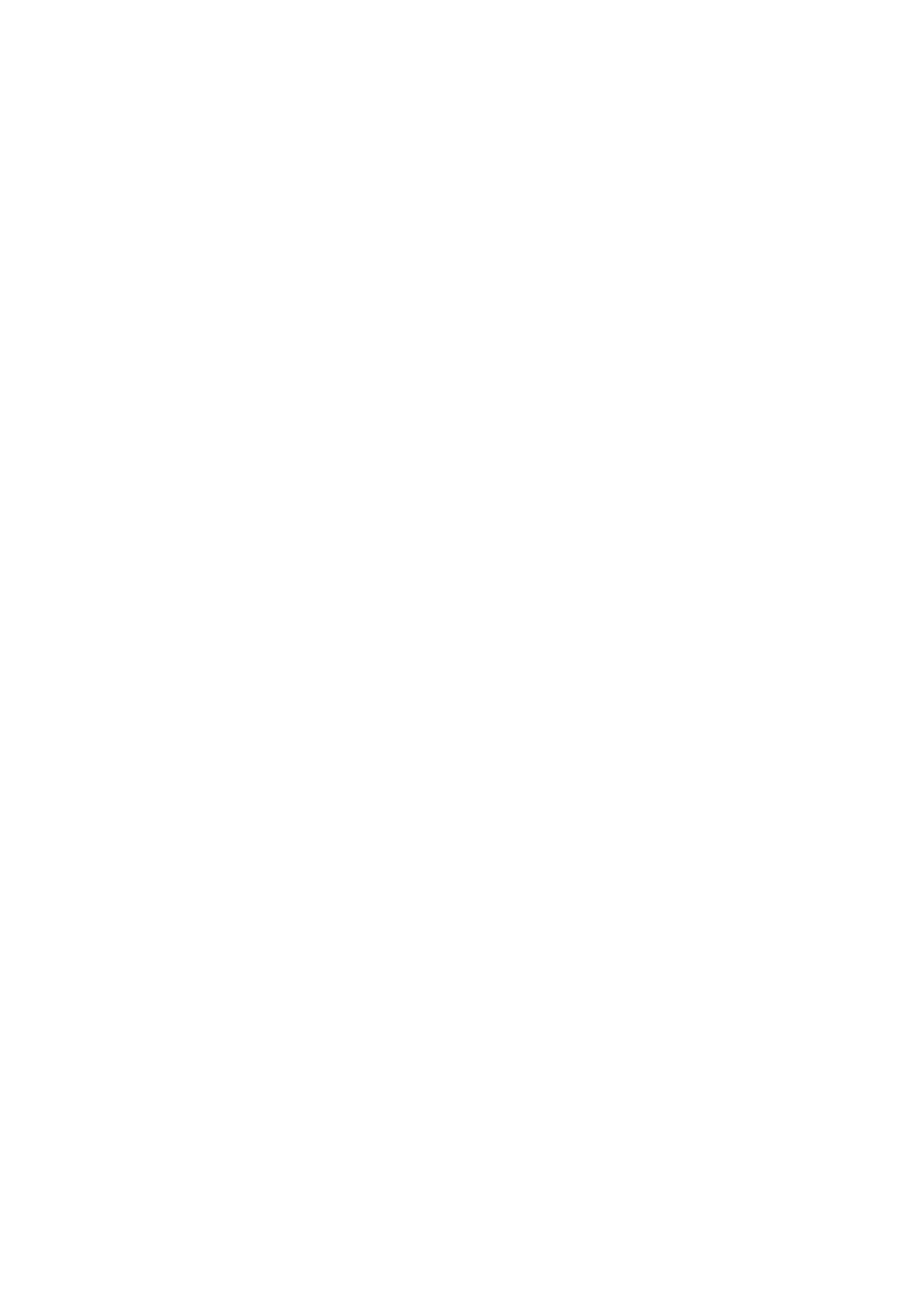

Supplement: Supplementary file 1 — Additional file 1 (PPTX 4505 KB) [file 12934_2025_2894_MOESM1_ESM.pptx]
