# Supplementary material for: Enhanced sucrose production by controlling carbon flux through CfrA expression in Synechocystis sp. PCC 6803
Source: Microb Cell Fact. 2025 Dec 31;25:29. doi: 10.1186/s12934-025-02894-8 (PMC12853614; doi:10.1186/s12934-025-02894-8)
Supplement: Supplementary file 2 — Additional file 2 (PPTX 52874 KB) [file 12934_2025_2894_MOESM2_ESM.pptx]

## Slide 1
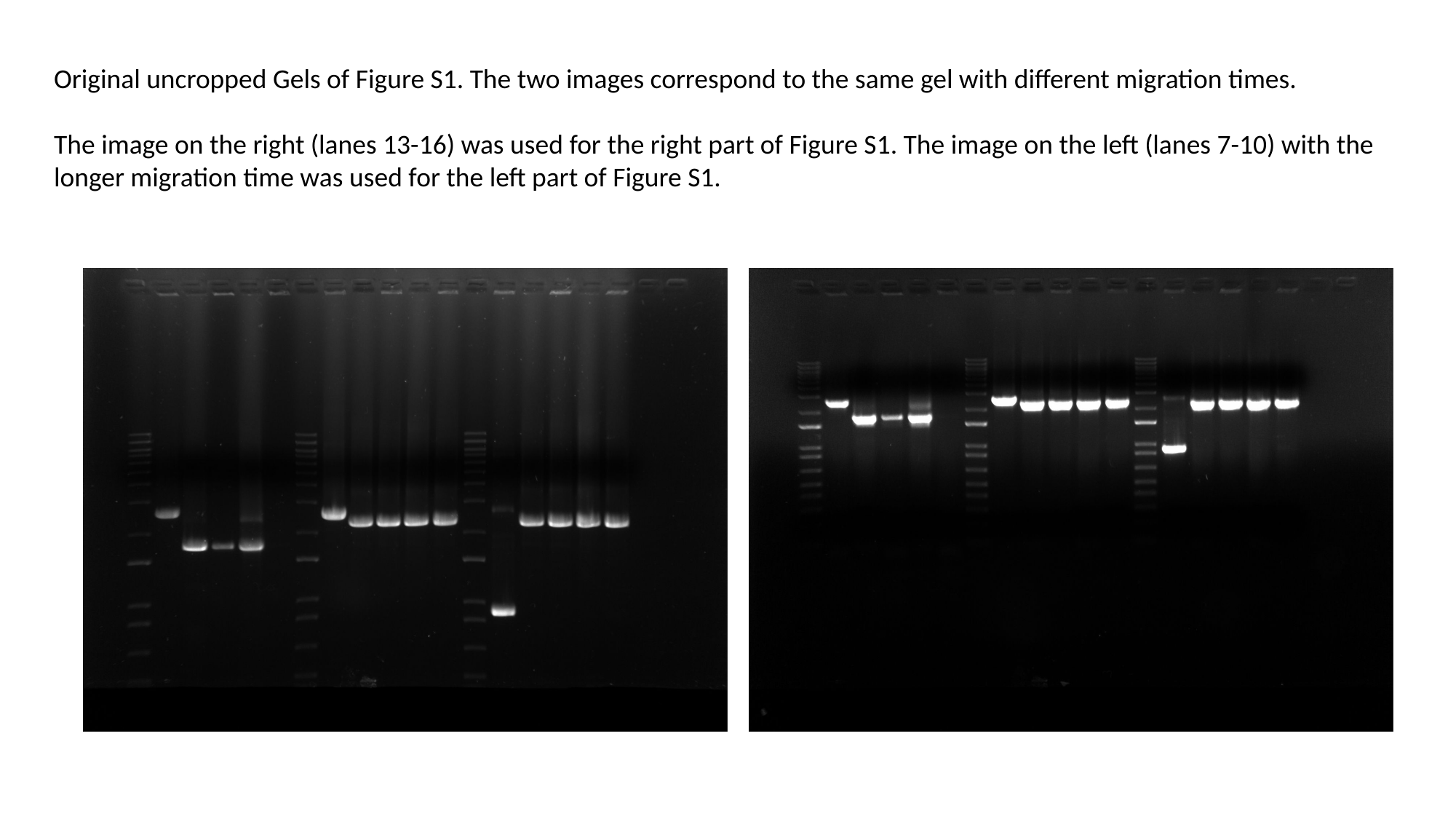

Original uncropped Gels of Figure S1. The two images correspond to the same gel with different migration times.
The image on the right (lanes 13-16) was used for the right part of Figure S1. The image on the left (lanes 7-10) with the longer migration time was used for the left part of Figure S1.
