# Supplementary material for: Enhanced sucrose production by controlling carbon flux through CfrA expression in Synechocystis sp. PCC 6803
Source: Microb Cell Fact. 2025 Dec 31;25:29. doi: 10.1186/s12934-025-02894-8 (PMC12853614; doi:10.1186/s12934-025-02894-8)
Supplement: Supplementary file 3 — Additional file 3 (PPTX 52873 KB) [file 12934_2025_2894_MOESM3_ESM.pptx]

## Slide 1
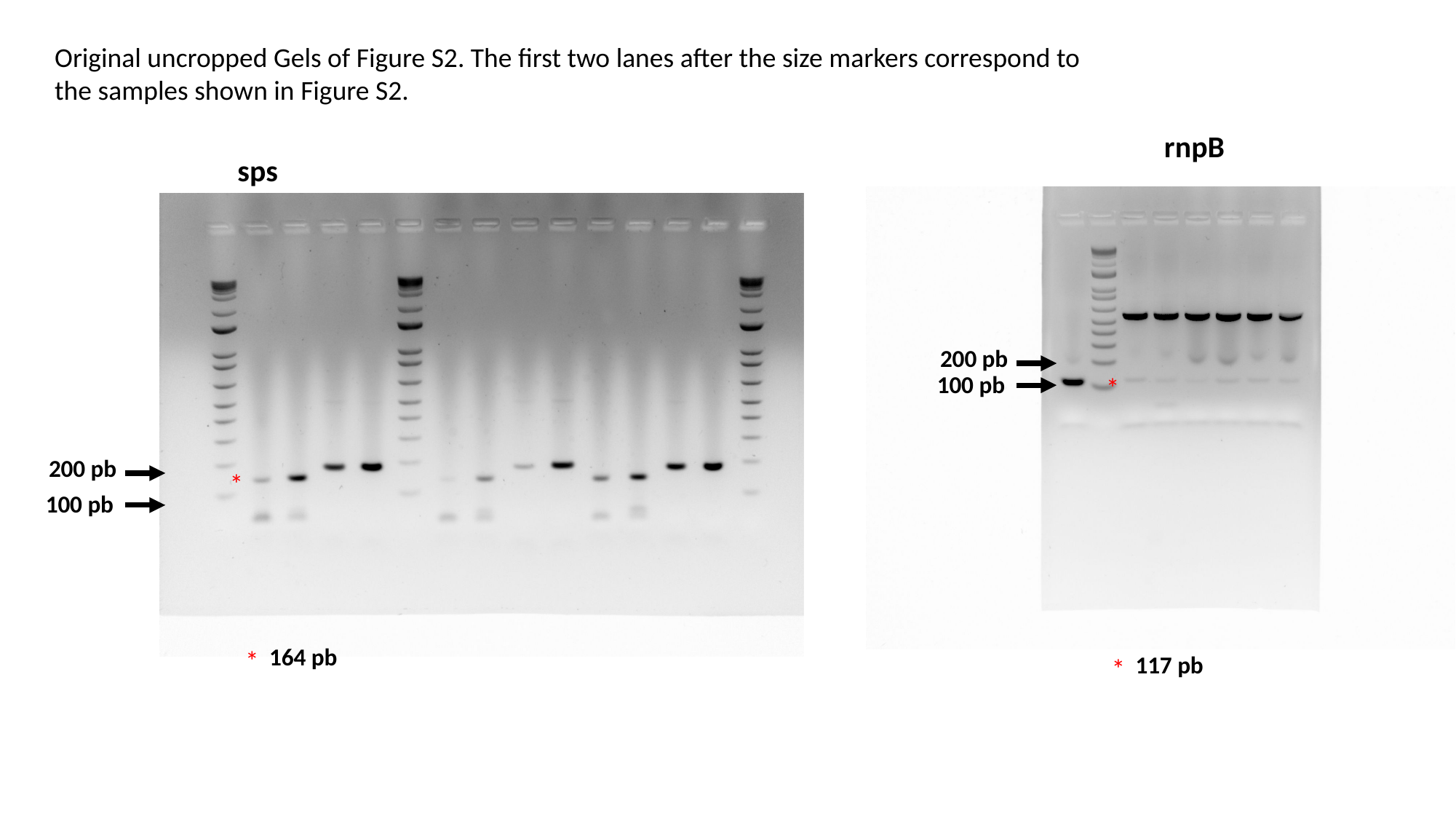

Original uncropped Gels of Figure S2. The first two lanes after the size markers correspond to the samples shown in Figure S2.
rnpB
 sps
 200 pb
 100 pb
*
 200 pb
*
 100 pb
 164 pb
*
 117 pb
*
