# Supplementary material for: Enhanced sucrose production by controlling carbon flux through CfrA expression in Synechocystis sp. PCC 6803
Source: Microb Cell Fact. 2025 Dec 31;25:29. doi: 10.1186/s12934-025-02894-8 (PMC12853614; doi:10.1186/s12934-025-02894-8)
Supplement: Supplementary file 4 — Additional file 4 (PPTX 435 KB) [file 12934_2025_2894_MOESM4_ESM.pptx]

## Slide 1
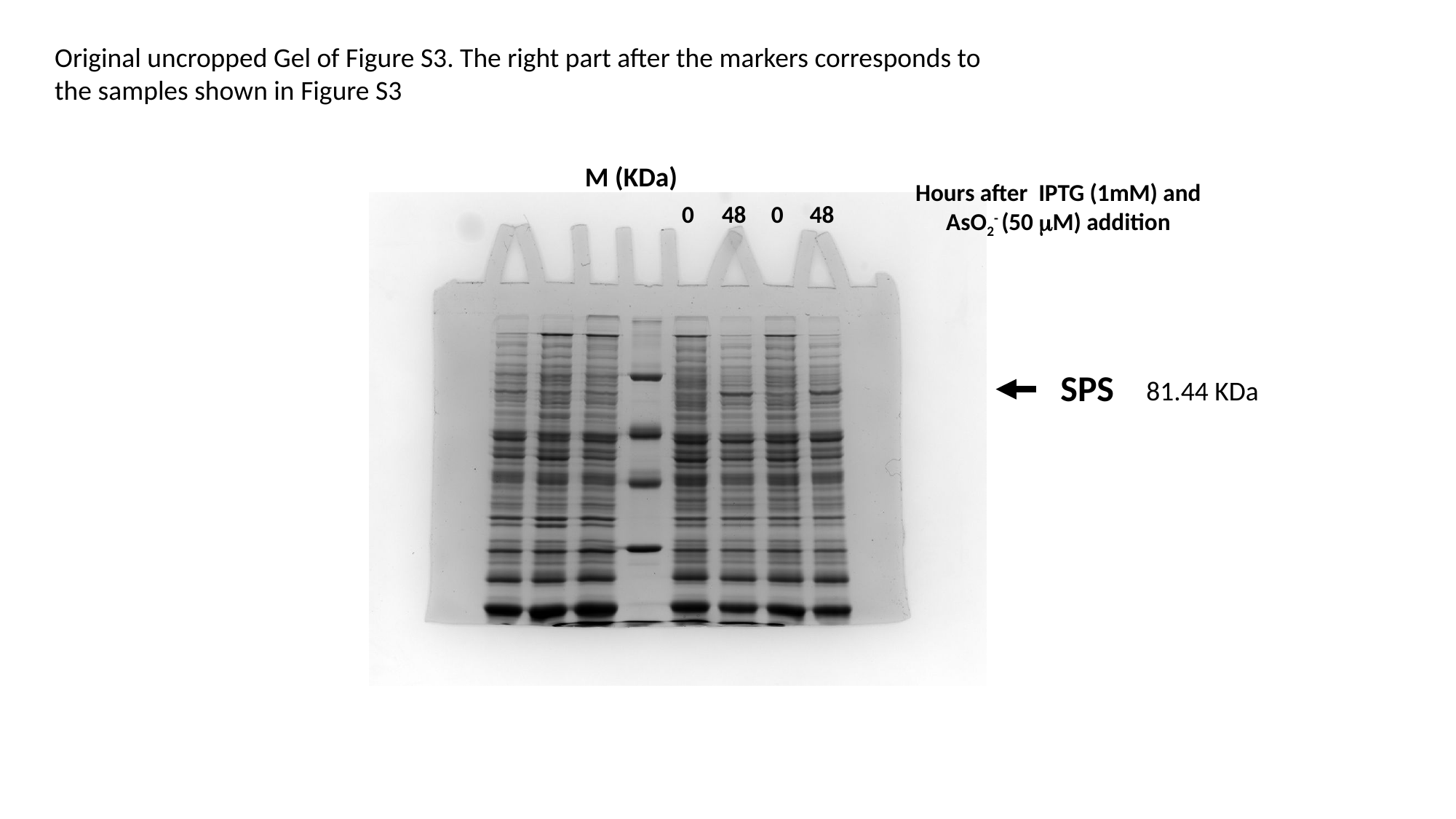

Original uncropped Gel of Figure S3. The right part after the markers corresponds to the samples shown in Figure S3
M (KDa)
Hours after IPTG (1mM) and AsO2- (50 mM) addition
0
48
0
48
SPS
81.44 KDa
